# Longitudinal analysis of coal workers’ pneumoconiosis using enhanced resolution-computed tomography images: unveiling patterns in lung structure, function, and clinical correlations

**DOI:** 10.3389/fphys.2025.1578058

**Published:** 2025-05-30

**Authors:** Ngan-Khanh Chau, Eun-Kee Park, Sanghun Choi

**Affiliations:** ^1^ School of Mechanical Engineering and IEDT, Kyungpook National University, Daegu, Republic of Korea; ^2^ An Giang University, Vietnam National University – Ho Chi Minh City, Ho Chi Minh, Vietnam; ^3^ Department of Preventive Medicine, College of Medicine, Kosin University, Busan, Republic of Korea

**Keywords:** coal workers with pneumoconiosis, longitudinal analysis, lung structure, lung function, clinical implications, disease progression

## Abstract

**Rationale:**

Pneumoconiosis, caused by prolonged exposure to mineral dust, leads to progressive structural and functional lung alterations. Quantitative computed tomography (qCT) has emerged as a critical tool for assessing these changes, yet there is limited research on the longitudinal patterns in pneumoconiosis patients.

**Methods:**

This study examined a cohort of 31 former coal workers with pneumoconiosis over a 1-year period. Inspiratory qCT images were enhanced using a deep learning-based super-resolution model and then processed to extract lung functional and airway structural metrics. A non-rigid image registration process was performed with baseline images as fixed and follow-up images as moving. Registration-derived metrics, including anisotropic deformation index (ADI), slab rod index (SRI), and Jacobian (J), were extracted to quantify regional deformation longitudinally. Pulmonary function tests, including forced expiratory volume in one second (FEV_1_) and forced vital capacity (FVC), were recorded at both time points to assess functional decline.

**Results:**

The study identified significant airway changes in angles, diameters, and geometry, with a decrease in normal lung tissue in the right upper lobe. Blood vessel volumes declined, indicating vascular remodeling. Registration metrics revealed regional heterogeneity, with higher ADI and SRI values and localized volume loss (J) in the lower lobes. FEV_1_/FVC progression correlated positively with tracheal angle, emphysema, and consolidation but negatively with normal lung tissue, semi-consolidation, and fibrosis. ADI, SRI, and J were associated with structural deformation, airway remodeling, and parenchymal loss, linking these changes to lung function decline.

**Conclusion:**

qCT imaging and registration metrics effectively monitor structural and functional lung changes in pneumoconiosis. Registering baseline and follow-up inspiration images offers additionally valuable insights into disease progression.

## 1 Introduction

Pneumoconiosis, a chronic occupational lung disease, is associated with prolonged exposure to mineral dust in industrial and working environments (Castranova and Vallyathan, 2000; Cullinan and Reid, 2013; Qi et al., 2021). It is predominantly caused by exposure to coal, silica, asbestos, and fine particulate matter, with inhaled particles depositing in the respiratory tract and triggering intricate inflammatory and fibrotic reactions in lung tissues. This condition gradually reduces lung function and overall quality of life, with persistent symptoms including cough, dyspnea, and fatigue. The pathogenesis involves a cascade of inflammatory responses triggered by deposited particles, which then lead to the release of profibrotic cytokines and the accumulation of fibrotic tissue. Subtypes of the disease include silicosis due to silica dust exposure, coal workers’ pneumoconiosis prevalent among coal miners, and asbestosis associated with asbestos inhalation. Despite efforts to safeguard workers from dust inhalation, pneumoconiosis remains a significant public health concern (Hoy and Chambers, 2020; Blanc and Seaton, 2016). The Global Burden of Disease study (Momtazmanesh et al., 2023) underscores the ongoing impact of pneumoconiosis, with 119,125 documented cases in the world in 2019. The number of deaths from the disease is also concerning, with 23,015 reported in the same year. The Disability-Adjusted Life Years reached a total of 919,077 years, reflecting the overall burden on health and wellbeing. This includes 479,340 years of life lost due to premature death associated with pneumoconiosis, and 439,737 years lived with disability.

Accurate assessment of pneumoconiosis requires advanced imaging techniques to evaluate the disease’s impact effectively. Traditional X-ray imaging has been widely used for identifying lung opacities and detecting pneumoconiosis-related changes (Blackley et al., 2018). However, it is still challenging to accurately evaluate the severity of the illness and track its progression using conventional approaches because of their subjectivity (Soydan, 2021). In recent years, quantitative computed tomography (qCT) has emerged as a promising tool for diagnosing pneumoconiosis (Xia et al., 2012). QCT offers precise three-dimensional visualization of lung structures and provides detailed information about the distribution, size, and characteristics of lesions (Akira et al., 2009). High-resolution CT and qCT have been shown to be useful diagnostic tools for detecting pneumoconiosis-related alterations and measuring disease severity (Rabiei et al., 2020). These advanced imaging techniques have the potential to enhance early detection, accurate assessment, and monitoring of pneumoconiosis, ultimately contributing to improved patient management and outcomes.

In our previous study, we conducted a cross-sectional analysis using inspiratory CT imaging to identify structural and functional imaging biomarkers associated with varying severities of coal workers’ pneumoconiosis (CWP) (Pyo et al., 2023). While that study provided important insights into airway alterations and parenchymal abnormalities, it was limited to single time-point assessments and inspiratory images alone. Building on these findings, the present study extends the investigation through a longitudinal approach, analyzing serial CT scans over a 1-year period to capture disease progression. Additionally, advanced imaging techniques such as a super-resolution generative adversarial network (GAN) and non-rigid image registration were applied to improve image quality and estimate functional deformation metrics, thus offering a more comprehensive evaluation of structural and functional changes over time.

The objective of this research is to understand the intricate factors driving the longitudinal progression of CWP, a debilitating lung disease caused by chronic exposure to mineral dust. By examining longitudinal data from CWP patients, this study aims to clarify the connections between structural changes, functional alterations, and clinical implications of the disease. A key aspect of the analysis involves leveraging a super-resolution GAN to enhance the quality of imaging data, allowing for the extraction of more precise and reliable quantitative metrics. Additionally, a non-rigid image registration was performed between inspiration images collected at baseline (fixed image) and at 1-year follow-up (moving image). This approach compensated for the absence of expiration images, enabling the estimation of deformation metrics and capturing both volumetric and directional changes in lung architecture over time. Through the investigation of correlations between functional and quantitative variables, this study seeks to illuminate the relationship between lung architecture, function, and clinical factors. Additionally, the focus is on understanding the relationship between tracheal geometry and functional parameters in order to identify markers of disease progression and potential intervention strategies.

## 2 Materials and methods

**FIGURE 1:**
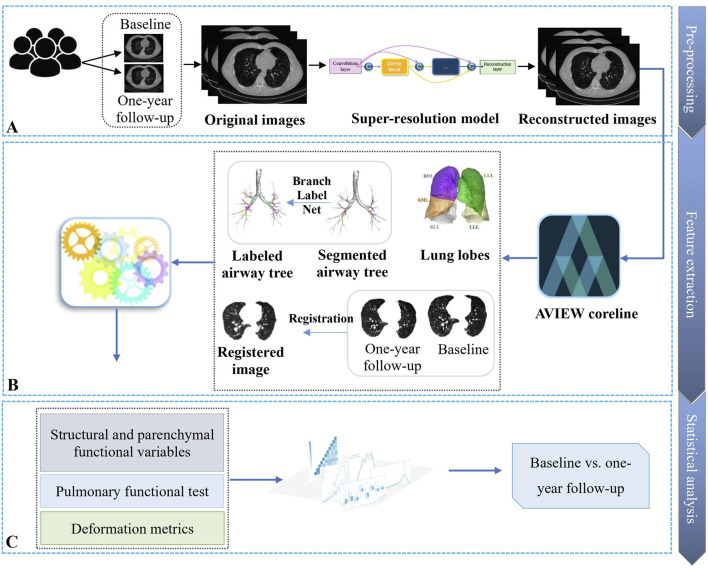
Overview of the workflow for analyzing the progression of coal workers’ pneumoconiosis. **(A)** First, the collected CT images are fed into a super-resolution model for image quality enhancement. **(B)** Next, qCT features are obtained from the reconstructed higher-resolution images. **(C)** Finally, statistical analysis is applied to the derived imaging features and clinical metrics to characterize disease progression patterns.

This section outlines our approach for analyzing the longitudinal progression in subjects with CWP. The methodology consists of three main steps: (1) pre-processing to enhance the CT image quality, (2) feature extraction to obtain airway structural and lung functional metrics, and (3) statistical analysis to gain insight into disease progression over time. Figure 1 depicts the workflow for this process.

### 2.1 Study population and image enhancement

A cohort of 31 CWP patients from Good Morning Hospital in South Korea were included in the study, with a 1-year gap between the two CT images taken (Pyo et al., 2023). Inclusion criteria for this subgroup required the availability of two inspiratory CT scans obtained approximately 1 year apart, without intervening thoracic surgery or major clinical events that could confound imaging interpretation. Patients without follow-up imaging or with significant imaging artifacts were excluded. In addition to imaging, all patients underwent spirometry-based pulmonary function testing at both time points. The measured parameters included forced expiratory volume in one second (FEV_1_), forced vital capacity (FVC), and their ratio (FEV_1_/FVC), which were used to assess longitudinal changes in lung function. Only total lung capacity (TLC) images were obtained for the analysis. The Siemens Somatom Scope scanner was used, with a spiral scan type and a rotation time of 1 s. The scanning protocol adhered to strict technical guidelines to ensure precision and reliability. The detector configuration was set at 16 × 1.2 mm, with a pitch value of 1.5 chosen for optimal coverage and image consistency. Radiation parameters were optimized using Care Dose modulation, and the B41s reconstruction algorithm with a slice thickness of 3 mm was used for image reconstruction.

These participants were drawn from the same cohort described in our previous study (Pyo et al., 2023), where detailed clinical profiles, including comorbidities and medication usage, were reported. In brief, patients were on a variety of medications commonly prescribed for CWP and associated respiratory conditions, such as bronchodilators, mucolytics, antihistamines, and oxygen therapy. No new medications or changes in treatment were introduced specifically for this longitudinal imaging study.

Due to the inherent limitations of CT image acquisition and storage protocols, the initial images were obtained at a relatively low-resolution. To enhance image quality for detailed analysis, we employed the deep learning-based super-resolution model developed by Chen et al. (2018), which effectively upscaled the images while preserving and enhancing fine anatomical details. This super-resolution pre-processing step was crucial for improving the visibility of subtle pathological changes and enabling a more accurate assessment of disease progression. Additional details about the super-resolution model can be found in the Supplementary Material.

### 2.2 QCT-based airway structural and parenchymal functional variables

Using AVIEW software (Corline Soft, Co., Ltd., Seoul, Republic of Korea) (Soft, 2024) and in-house post-processing methods, airway structural characteristics were retrieved from CT scans. Following the methodology proposed in Choi et al. (2015), we derived the one-dimensional (1D) airway skeleton and calculated key structural variables, such as luminal area (LA), luminal area perimeter (P_e_), and wall thickness (WT), from the TLC scans. Airway branches were identified using BranchLabelNet, an in-house deep learning model developed in our previous study (Chau et al., 2024) for automated human airway branch labeling. All labeling results were manually verified for accuracy.

**FIGURE 2:**
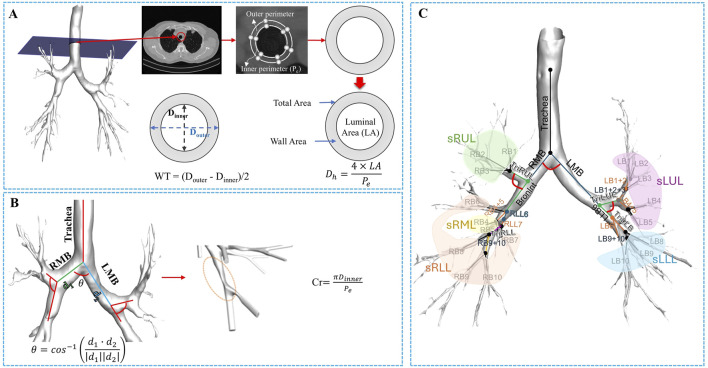
The procedure of extracting structural variables from a segmented airway image. **(A)** From the 1D skeleton airway, the luminal area (LA), wall area, and total area were extracted. The hydraulic diameter (D_h_) was then calculated by associating the LA and the inner perimeter (P_e_), followed by determining the wall thickness (WT) as the difference between the luminal average diameter (D_inner_) and the outer diameter (D_outer_). **(B)** From the segmented airway, the bifurcation angle (θ) was calculated, and circularity was used to assess the degree of non-circularity. **(C)** The illustration of the segmental airways, such as the trachea, left and right main bronchus (LMB and RMB), Bronint, four trifurcation branches, and five lobar subgroups.

Additionally, bifurcation angle (θ), circularity (Cr), and hydraulic diameter (D_h_) were obtained from the dataset (Choi et al., 2015). The process for extracting these variables is illustrated in Figure 2. As illustrated in Figure 2, bifurcation angle was computed using the cosine law based on the vectors of adjoining branches at a bifurcation: $\theta =\cos^{-1}\left(\frac{d_{1}\cdot d_{2}}{\left|d_{1}\right|\left|d_{2}\right|}\right)$ , where $d_{1}$ and $d_{2}$ represent the direction vectors of daughter branches. This angle characterizes the branching pattern of the airway and may reflect developmental or disease-induced structural changes. Circularity was defined as $Cr=\frac{\pi D_{inner}}{P_{e}}$ , indicating how closely the airway cross-section approximates a perfect circle (Cr = 1 for a circle). It is influenced by both luminal shape and potential structural deformation. Hydraulic diameter was calculated as $D_{h}=\frac{4\times LA}{P_{e}}$ . This metric reflects airway cross-sectional size and shape and is personalized for each subject, depending on their specific airway geometry. To quantify branch-level airway structure, measurements of θ, D_h_, WT, and Cr were extracted from the trachea, main bronchi, intermediate bronchus (Bronint), and four lobe-specific trifurcation points, as depicted in Figure 2C. Additionally, D_h_, WT, and Cr were calculated across five lobe-defined airway subgroups to capture localized structural variations.

Furthermore, to reduce the potential impact of confounding factors such as age, sex, and height, we applied a normalization technique based on multiple linear regression models developed in the study (Chae et al., 2021). Specifically, reference values for tracheal diameter (pD_trachea_), tracheal LA (pLA_trachea_), and tracheal WT (pWT_trachea_) were predicted as the following equations:

$${\text{pD}}_{\text{trachea}}\,\left(\text{mm}\right)=12.79\;–\;0.13\log\left(\text{age}\right)\;–\;5.82\log\left(\text{height}\right)*\text{sex}+3.01\log\left(\text{age}\right)*\log\left(\text{height}\right)$$

$${\text{pLA}}_{\text{trachea}}\,\left({\text{mm}}^{2}\right)=122.23\;–\;5.45\log\left(\text{age}\right)\;–\;148.13\log\left(\text{height}\right)*\text{sex}+77.75\log\left(\text{age}\right)*\log\left(\text{height}\right)$$

$${\text{pWT}}_{\text{trachea}}\,\left(\text{mm}\right)=\log\left[9.11\;–\;1.02\log\left(\text{age}\right)\;–\;0.98\,{\text{height}}^{2}*\text{sex}+1.01\,{\text{height}}^{2}*\log\left(\text{age}\right)\right]$$

where sex was represented as a binary variable (0 = male, 1 = female), and height and age were recorded in meters and years, respectively.

These equations accounted for individual age, sex, and height. Each subject’s airway measurements (D_h_, LA, and WT) were then normalized by dividing them by their respective predicted reference values (pD_trachea_, pLA_trachea_, pWT_trachea_). This approach effectively controls for inter-subject variability and enables more accurate comparisons across individuals. These metrics characterize various features of airway and lung geometry: wall thickness, luminal area, perimeter, and circularity provide insight into airway dimensions and morphology, while bifurcation angle and hydraulic diameter shed light on branching patterns and flow dynamics within the pulmonary system.

Moreover, we multiplied the ratio of the average to the individual scan interval to adjust the variable’s progression and mitigate the impact of the interval (Kim et al., 2022). The progression metric of the variable is represented by the following equation:

$$progression=\frac{{var}_{follow-up}-{var}_{baseline}}{{var}_{baseline}}\left(\frac{average\;scan\;interval}{individual\;scan\;interval}\right)$$

In this equation, ${var}_{baseline}$ and ${var}_{follow-up}$ denote the values of a variable at baseline and at 1-year follow-up, respectively. Individual scan interval represents the interval of each subject, and the average scan interval is 360.10 days in our study. This correction assumes a relatively consistent rate of disease progression across individuals, which we considered reasonable given the narrow distribution of follow-up intervals in our cohort.

**FIGURE 3:**
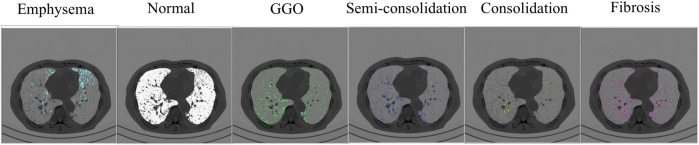
The severity of parenchymal abnormalities in subjects is determined by CT Hounsfield units. The visualization demonstrates multiple lung states, spanning from emphysematous regions to normal parenchyma, areas of semi-consolidation and consolidation, and fibrotic changes.

Functional variables encompass distinct indices designed to measure pertinent aspects of pulmonary dynamics. These variables consist of a ratio based on CT Hounsfield unit (HU) to quantify anomalous regions and blood vessel volume metrics based on the ratio of pulmonary vessels’ cross-sectional area. The identification of abnormal regions, accomplished through the application of HU thresholds, includes a spectrum of distinct pathological states: emphysema (HU below −950) (Matsuoka et al., 2015), normal lung (HU ranging from −950 to −701) (Colombi et al., 2020), ground-glass opacity (GGO, HU spanning from −700 to −501) (Liu et al., 2020), semi-consolidation (HU between −500 and −201) (Liu et al., 2020), consolidation (HU spanning from −200 to 60) (Liu et al., 2020), and fibrosis (HU ranging from −500 to 0) (Matsuoka et al., 2015). These delineations are based on previous studies, which establish their clinical significance. Figure 3 visualizes the emphysema, normal lung, ground-glass opacity, semi-consolidation, consolidation, and fibrosis in a subject at baseline.

The blood vessel volumes were derived using the AVIEW software (Soft), which included the assessment of total blood vessel volume (TBV) and the volume of blood vessels (BV) through cross-sectional areas ranging from less than 1 mm^2^ to less than 20 mm^2^ (from BV1 to BV20). These quantitative indicators precisely represent the level of pulmonary vascular constriction in the research context.

To assess longitudinal lung deformations, a non-rigid image registration process was performed using the B-spline registration algorithm (Rueckert et al., 1999) implemented in the ITK library (Avants et al., 2014). In this approach, the baseline lung image served as the fixed image, while the 1-year follow-up lung image was designated as the moving image. The B-spline registration algorithm was chosen for its ability to model complex, non-linear deformations with high accuracy and flexibility. Following the registration process, deformation metrics including the anisotropic deformation index (ADI), slab rod index (SRI), and Jacobian (J) were extracted from various lung regions (Choi et al., 2013). The J was computed to quantify local volume changes, identifying areas of expansion or contraction within the lung. The ADI measured the magnitude of directional preference in volume changes, providing insight into the anisotropic nature of the deformation. The SRI was calculated to characterize the specific directional preference in volume change, distinguishing between slab-like and rod-like deformations. Together, these metrics enabled a comprehensive evaluation of both the magnitude and directional patterns of regional lung deformation over the 1-year period.

### 2.3 Statistical methods

Initially, we assessed data normality using the Shapiro-Wilk test (Shapiro and Francia, 1972). Data that followed a normal distribution underwent paired t-test analysis (Rosner, 1982), while the Wilcoxon paired test (Wilcoxon, 1992) was employed for non-normal distributions. We set the significance level at p < 0.05 to determine statistical relevance for both tests. To uncover relationships between functional and quantitative variables, Spearman’s correlation coefficients (Wissler, 1905) were calculated at both baseline and the 1-year follow-up. The correlational scale spans from −1 to +1, with the negative limit indicating complete inverse correspondence and the positive limit showing complete direct correspondence. This multifaceted statistical approach allowed for an in-depth examination of the complex longitudinal aspects of CWP progression, advancing insights in this field. The statistical analyses were conducted using the stats package in Python 3.11.4.

## 3 Results

### 3.1 Demographic information

Table 1 provides a detailed description of the demographic data for the longitudinal cohort consisting of 31 subjects with CWP (N = 31). The average scan interval between the baseline and the 1-year follow-up was 360.10 (standard deviation, 15.38) days. Analysis of pulmonary functional test (PFT) measurements revealed that the overall lung function of the subjects remained relatively stable over the 1-year period. However, it is noteworthy that a significant decline in FEV_1_ was observed at the 1-year follow-up, indicating a gradual decline in lung function. Smoking status was determined based on self-reported history at the time of enrollment. Participants were classified as smokers if they had smoked at least 100 cigarettes in their lifetime, following the standard epidemiological definition established by the Centers for Disease Control and Prevention (CDC).

**TABLE 1:** Demographic and PFT data for individuals with pneumoconiosis at baseline and after 1 year. Values are displayed as means, followed by standard deviation in parentheses.

N = 31	Baseline	One-year follow-up	p-value
Interval, days		360.10 (15.38)	-
Male/Female, n (%)	30/1 (96.77%/3.23%)	-	-
Smoking status, n (%)	28 (90.32%)	-	-
Age, years	75.19 (7.12)	76.71 (7.07)	-
Height, cm	162.81 (5.74)	-	-
Weight, kg	60.26 (7.98)	-	-
BMI, kg/m^2^	22.75 (2.9)	-	-
FEV_1_, %	49.23 (20.5)	45.69 (16.12)	**<0.05**
FVC, %	54.71 (13.84)	54.27 (12.05)	0.303
FEV_1_/FVC, %	55.92 (16.68)	55.52 (18.17)	0.346
Lung volume (cc)			
Whole lung	4143 (1102)	4017 (1005)	0.126
Right upper lobe	725 (398)	711 (391)	0.424
Right middle lobe	319 (195)	294 (189)	0.193
Right lower lobe	1142 (337)	1113 (333)	0.324
Left upper lobe	952 (400)	920 (395)	0.082
Left lower lobe	1005 (364)	979 (338)	0.338

BMI, body mass index; FEV_1_, forced expiratory volume in one second; FVC, forced vital capacity. The bold value indicates a statistically significant difference (p-value < 0.05).

To address the limitations of low-resolution imaging, we implemented a modified version of the super-resolution model originally proposed by Chen et al. (2018). This enhancement procedure was performed prior to qCT feature extraction. Additional information on the image reconstruction process can be found in the Supplementary Material.

### 3.2 Airway structural variables analysis

Structural and quantitative changes in individuals with CWP (N = 31) over a 1-year period were analyzed, focusing on key parameters such as branching angle (θ), hydraulic diameter (D_h_), and wall thickness (WT). No significant changes were observed in the main bronchial branches, including the trachea and primary airways, during the study period. However, alterations were noted in smaller airway branches, primarily involving secondary (lobar bronchi) and tertiary (segmental bronchi) regions. These branches correspond to the airways supplying each lung lobe, such as the right upper lobe bronchus, right middle lobe bronchus, left upper lobe bronchus, and their respective segmental subdivisions. Notable changes in branching angle, hydraulic diameter, wall thickness, and circularity were observed in these regions, indicating localized structural remodeling. A summary of significant findings is provided in Table 2, with complete statistics available in Supplementary Table S3.

**TABLE 2:** The qCT-based airway structural measurements were obtained from baseline and 1-year follow-up images.

Region	Baseline	One-year later	p-value	Q-value
Angle, θ				
TriRLL	67.555 (27.47)	54.54 (36.21)	0.01	0.012
LB4+5	97.224 (34.52)	77.587 (34.39)	0.005	0.009
LB9+10	48.365 (24.43)	63.569 (32.27)	0.02	0.04
LB3	72.407 (30.7)	65.984 (27.7)	0.026	0.032
LB4	77.279 (25.53)	94.9 (23.96)	0.000004	0.000008
LB5	80.181 (38.07)	108.828 (35.32)	0.000003	0.000006
RB9+10	70.479 (36.27)	93.724 (42.07)	0.005	0.011
RB2	76.531 (37.93)	65.423 (24.02)	0.019	0.028
RB3	75.07 (27.52)	89.258 (34.47)	0.000495	0.000991
RB4	58.086 (23.47)	72.085 (23.12)	0.000045	0.000067
RB5	92.647 (38.92)	69.981 (40.61)	0.000087	0.000173
RB6	62.852 (29.17)	82.207 (36.14)	0.000414	0.000828
RB10	69.437 (35.92)	81.238 (39.95)	0.019	0.037
Hydraulic diameter, D_h_				
TriLLB	8.539 (1.92)	9.466 (2.42)	0.018	0.035
LB1+2	4.792 (1.59)	5.578 (1.87)	0.013	0.026
LB2	4.061 (1.67)	4.57 (1.45)	0.011	0.031
LB3	5.933 (3.61)	7.433 (4.98)	0.03	0.043
Wall thickness, WT				
LB1+2 + 3	5.447 (0.9)	5.868 (0.86)	0.028	0.036
RB8	4.1 (1.22)	3.451 (0.93)	0.001	0.002
RB9	3.654 (0.81)	3.297 (0.87)	0.021	0.043
Circularity, Cr				
LB6	0.921 (0.05)	0.891 (0.05)	0.017	0.034
RB2	0.943 (0.03)	0.923 (0.04)	0.029	0.059
RB4	0.909 (0.07)	0.926 (0.06)	0.048	0.052
RB8	0.918 (0.06)	0.955 (0.03)	0.0007	0.0014

θ, bifurcation angle; D_h_, hydraulic diameter; WT, wall thickness; Cr, circularity; TriLLB, trifurcation of the left lower lobe; TriRLL, trifurcation of the right lower lobe. Values are displayed as means with standard deviations provided in parentheses.

### 3.3 Parenchymal functional variables analysis

Table 3 presents a detailed analysis of functional variables, revealing dynamic changes in parenchymal characteristics associated with the progression of pneumoconiosis. The results cover a variety of functional metrics, including the proportions of consolidation (Conso), semi-consolidation (Semiconso), normal lung tissue (Norm), emphysema (Emph), ground-glass opacity (GGO), and fibrosis (Fibr) in various lung regions.

**TABLE 3:** Parenchymal function metrics were extracted from baseline and 1-year follow-up images using qCT.

Region	Baseline	One-year later	p-value	Q-value
Consolidation, Conso (%)				
LUL	2.779 (1.64)	2.988 (1.71)	0.357	0.468
LLL	2.365 (1.1)	2.281 (1.01)	0.568	0.666
RUL	2.859 (1.33)	3.237 (1.53)	0.055	0.176
RML	1.995 (1.53)	2.009 (1.39)	0.568	0.753
RLL	2.595 (1.41)	2.592 (1.14)	0.327	0.364
Total	2.513 (1.06)	2.605 (1.05)	0.202	0.315
Semi-consolidation, Semiconso (%)				
LUL	5.479 (3.53)	5.385 (3.23)	0.75	0.939
LLL	8.191 (8.08)	6.542 (3.45)	0.636	0.762
RUL	5.731 (3.35)	5.482 (3.16)	0.721	0.81
RML	4.99 (3.51)	4.141 (2.36)	0.839	0.929
RLL	8.37 (7.86)	6.853 (4.01)	0.542	0.653
Total	6.539 (4.94)	5.578 (2.6)	0.9	0.989
Normal, Norm (%)				
LUL	58.012 (25.17)	55.457 (27.51)	0.433	0.504
LLL	59.773 (21.69)	61.446 (18.81)	0.824	0.881
RUL	**58.775 (26.25)**	**55.001 (28.85)**	**0.037**	**0.038**
RML	62.55 (25.23)	59.713 (26.91)	0.75	0.938
RLL	61.552 (21.55)	61.55 (18.09)	0.347	0.434
Total	60.576 (21.72)	59.078 (22.35)	0.141	0.199
Emphysema, Emph (%)				
LUL	7.707 (11.3)	8.035 (11.98)	0.399	0.531
LLL	4.859 (6.47)	4.792 (7.19)	0.29	0.446
RUL	5.89 (8.45)	5.694 (8.76)	0.29	0.429
RML	9.344 (10.76)	10.069 (14.49)	0.794	0.875
RLL	4.057 (6.64)	4.404 (7.67)	0.706	0.782
Total	6.016 (7.72)	6.025 (8.32)	0.239	0.243
Ground-glass opacity, GGO (%)				
LUL	10.269 (7.56)	9.37 (6.69)	0.961	1
LLL	14.813 (10.67)	13.14 (7.97)	0.961	0.995
RUL	10.516 (7.36)	9.565 (6.29)	0.664	0.796
RML	8.374 (6.12)	7.174 (4.83)	0.915	0.971
RLL	14.56 (10.17)	13.959 (9.17)	0.839	0.893
Total	12.011 (8.14)	10.909 (6.67)	0.915	0.964
Fibrosis, Fibr (%)				
LUL	7.664 (4.27)	7.925 (3.85)	0.209	0.29
LLL	9.512 (8.96)	7.709 (4.01)	0.468	0.565
RUL	8.187 (3.58)	8.303 (3.7)	0.232	0.378
RML	7.654 (4.95)	8.236 (5.52)	0.347	0.434
RLL	10.435 (8.97)	9.031 (4.88)	0.247	0.397
Total	8.552 (5.49)	7.767 (2.81)	0.636	0.767

LUL, left upper lobe; LLL, left lower lobe; RUL, right upper lobe; RML, right middle lobe; RLL, right lower lobe; Conso, consolidation; Semiconso, semi-consolidation; Norm, normal; Emph, emphysema; GGO, ground-glass opacity; Fibr, fibrosis. The bold value indicates a statistically significant difference (p-value < 0.05).

**FIGURE 4:**
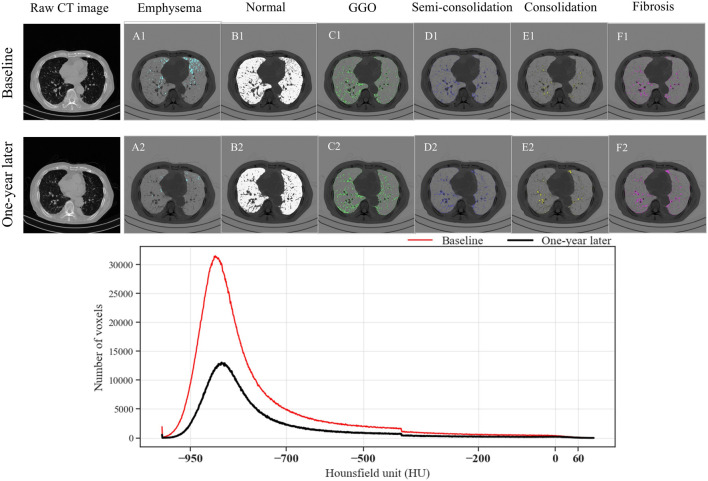
Comparison of parenchymal characteristics of a randomly selected subject at baseline and 1 year later. **(A1–F1)** The images show emphysema, normal lung, ground-glass opacity, semi-consolidation, consolidation, and fibrosis at baseline, and **(A2–F2)** at the 1-year follow-up. The line chart presents the number of voxels in each Hounsfield unit category, segmented from the lung region. The total number of voxels reflects the volume and density distribution of lung tissue; a reduction over time may suggest parenchymal loss or disease progression.

An evaluation of the data revealed notable patterns. For example, consolidation measurements remained relatively stable across lung lobes, with only minor variations in the overall lung percentages. Likewise, semi-consolidation metrics showed modest fluctuations, with lobar and total percentages indicating a degree of consistency. Normal lung tissue compositions experienced minimal changes, with slight deviations observed in both regional and overall metrics. In contrast, emphysema and GGO percentages demonstrated distinct patterns. Emphysema percentages exhibited limited fluctuations across the lung lobes and the entire lung, while GGO percentages remained relatively stable across both lobes and the total lung. Fibrosis percentages, analyzed across various lung regions, showed consistent trends, reflecting stability throughout the entire lung, the right and left lungs, and their subdivisions. An exception to these variables was the normal lung tissue in the right upper lobe (RUL), which experienced a significant change after 1 year with a p-value below 0.05. Additionally, over the course of a year, a significant reduction in the total voxel count was observed (see Figure 4). This total voxel count represents the number of CT image voxels within the segmented lung region that fall within the defined HU range of −1000 to 0, which reflects aerated lung tissue. A reduction in total voxel count may indicate volume loss, tissue consolidation, or progression of fibrotic changes.

**FIGURE 5:**
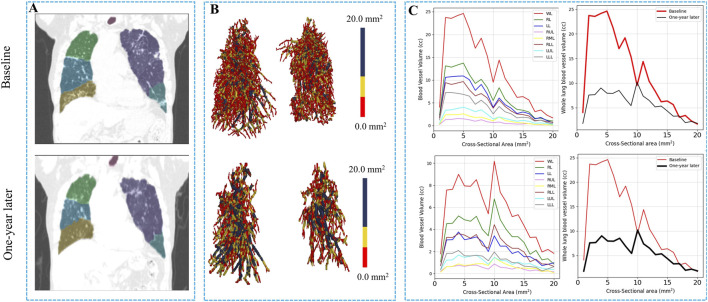
Blood vessel volume comparison in one randomly selected subject at baseline and 1 year later. **(A)** The images of the subject at baseline and 1-year later. **(B,C)** Reconstructions of the pulmonary blood vessels in volume of the subject. The volume of blood vessels in the cross-sectional region of the subject ranging from 1 mm^2^ to 20 mm^2^ according to each region and the whole lung. WL, whole lung; RL, right lung; LL, left lung; LUL, left upper lobe; LLL, left lower lobe; RUL, right upper lobe; RML, right middle lobe; RLL, right lower lobe.

**FIGURE 6:**
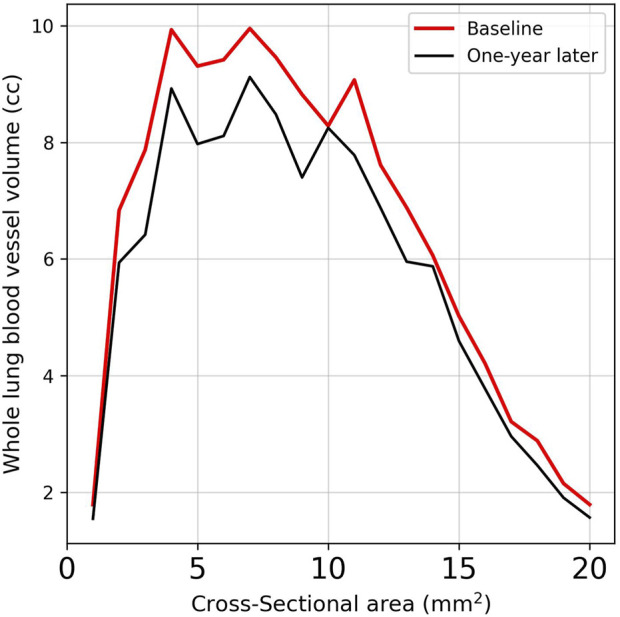
Comparison of the average vessel volumes measured across cross-sectional areas in subjects using images obtained at baseline and 1-year follow-up.

Table 4 provides a comprehensive analysis of blood vessel-related functional variables in the context of pneumoconiosis. These variables are measured across various blood vessel cross-sectional areas, labeled as BV1 through BV20 from 1 mm^2^ to 20 mm^2^, in addition to the total blood volume (TBV). The data shows that most of the blood vessel cross-sectional area variables exhibit marginal fluctuations between baseline and 1-year follow-up assessments. Of particular interest, the TBV, a crucial indicator of overall blood vessel volume, exhibited a statistically significant difference (p < 0.05). As a result, at the 1-year follow-up, the pulmonary vessel cross-sectional area ratio was narrower compared to baseline. This reduction was consistently significant across multiple metrics (Supplementary Table S4). Specifically, the left upper lung (LUL) showed significant reductions in subjects from BV11 through BV20, with p-values ranging from 0.036 to 0.048. Significant decreases were also observed in the total blood vessel volumes of the left lung and the whole lung, both with p-values of 0.031. Figure 5B visualizes the blood vessels of a randomly selected participant (Figure 5A), while Figure 5C depicts the volume of blood vessels in each cross-sectional region ranging from 1 mm^2^ to 20 mm^2^. The mean values for all participants are shown in Figure 6.

**TABLE 4:** Blood vessel volume in the entire lung extracted from baseline and 1-year follow-up images.

Variable	Baseline	One-year later	p-value	Q-value
BV1	1.788 (1.43)	1.547 (1.02)	0.299	0.308
BV2	8.622 (7.09)	7.485 (5.05)	0.597	0.608
BV3	16.494 (11.98)	13.902 (8.23)	0.232	0.239
BV4	26.423 (18.46)	22.824 (13.88)	0.357	0.367
BV5	35.729 (23.5)	30.795 (17.98)	0.327	0.337
BV6	45.144 (28.53)	38.904 (22.19)	0.189	0.195
BV7	55.094 (33.62)	48.022 (27.02)	0.337	0.347
BV8	64.55 (37.75)	56.5 (30.94)	0.183	0.189
BV9	73.369 (41.51)	63.898 (33.75)	0.131	0.135
BV10	81.651 (44.56)	72.145 (37.43)	0.147	0.152
BV11	90.72 (48.52)	79.926 (40.8)	0.147	0.152
BV12	98.333 (50.77)	86.803 (42.59)	0.1	0.102
BV13	105.214 (53.28)	92.757 (44.63)	0.075	0.076
BV14	111.276 (55.13)	98.631 (46.51)	0.1	0.102
BV15	116.297 (56.75)	103.226 (47.96)	0.092	0.094
BV16	120.506 (57.93)	107.001 (49.01)	0.096	0.098
BV17	123.718 (58.73)	109.96 (49.72)	0.085	0.087
BV18	126.601 (59.51)	112.422 (50.54)	0.065	0.066
BV19	128.753 (60.18)	114.33 (50.89)	0.055	0.055
BV20	130.544 (60.58)	115.898 (51.29)	0.055	0.055
TBV	**138.332 (61.95)**	**123.08 (52.68)**	**0.031**	**0.043**

BV, blood vessel volume; TBV, total blood volume. The bold value indicates a statistically significant difference (p-value < 0.05).

**FIGURE 7:**
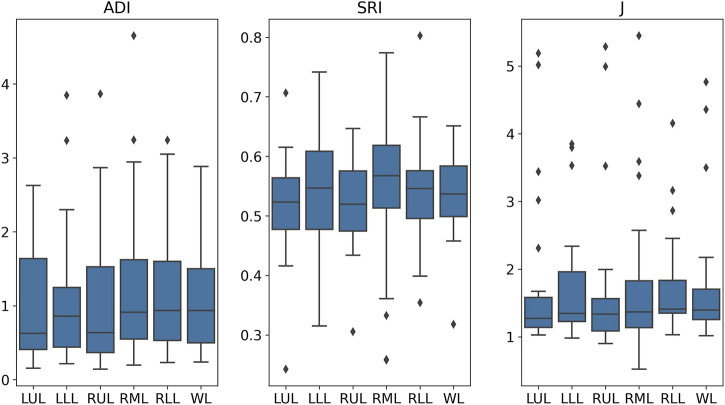
Distributions of ADI, SRI, and J values across various lung regions. WL, whole lung; RL, right lung; LL, left lung; LUL, left upper lobe; LLL, left lower lobe; RUL, right upper lobe; RML, right middle lobe; RLL, right lower lobe.

**FIGURE 8:**
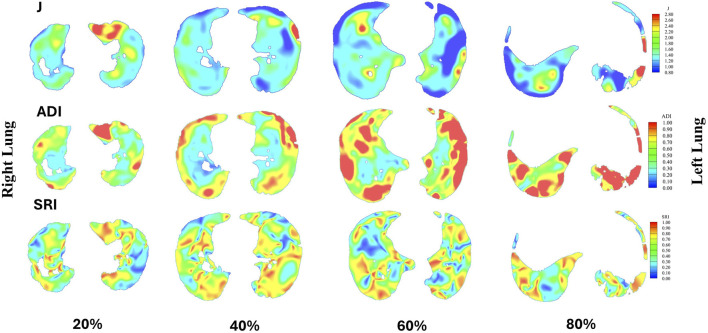
Distributions of J, ADI, and SRI for a sample subject are presented at 20% (near apex), 40%, 60%, and 80% (near base) from the apical to basal regions. In each slice, the right lung is shown on the left, and the left lung is shown on the right.

The analysis of anatomical and volumetric changes over a 1-year period, as captured by the ADI, SRI, and J, revealed significant regional heterogeneity within the lungs (see Figure 7). Notably, the lower lobes, including the LLL and RLL, exhibited higher median deformation (ADI) and SRI values compared to the upper lobes, indicating more pronounced structural changes over time. Similarly, the J value also highlighted greater contraction in these regions, suggesting localized volume loss. In contrast, the upper lobes, particularly the RUL, displayed lower variability and smaller magnitudes of deformation. The whole lung (WL) metrics, while reflecting a moderate overall change, masked substantial regional differences, underscoring the importance of localized analysis in understanding longitudinal lung deformations. Figure 8 shows the distributions of one sample subject. J demonstrated smaller values in the lower (80%, near base) compared to the upper (20%, near apex); SRI exhibited a similar decreasing trend as ADI from the lower to the upper, though with less pronounced changes.

### 3.4 Correlation analysis

**FIGURE 9:**
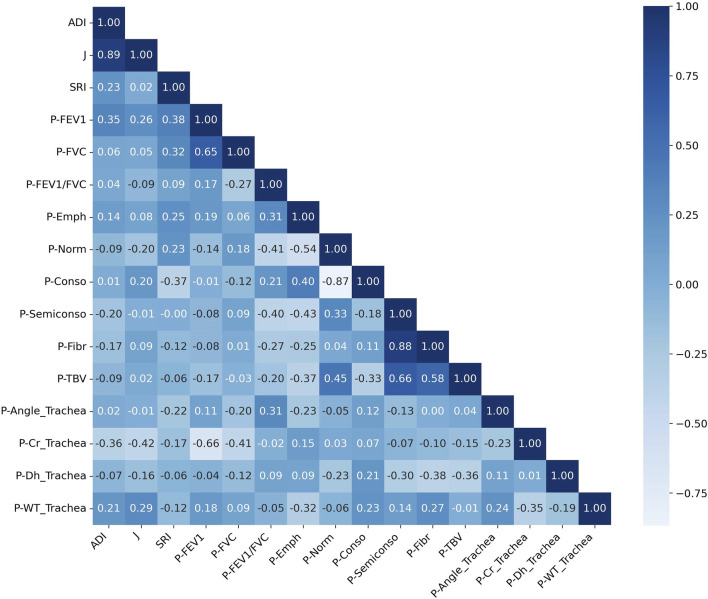
Correlation matrix of changes in structural and functional variables over a 1-year interval. A positive correlation is shown by values close to 1, a negative correlation by values close to −1, and no correlation by values close to 0. The prefix “P” denotes the “progression”. ADI, anisotropic deformation index; SRI, slab rob index; J, Jacobian; FEV1, forced expiratory volume in one second; FVC, forced vital capacity; Cr, circularity; Dh, hydraulic diameter; WT, wall thickness; Emph, emphysema; Norm, normal; Conso, consolidation; Semiconso, semi-consolidation; Fibr, fibrosis; TBV, total blood volume.

The Spearman correlation analysis revealed significant relationships between the progression of pulmonary function, tracheal structural metrics, and lung pathology indicators, suggesting complex interactions among these aspects (Figure 9). The progression of FEV_1_ (P-FEV1) exhibited a strong negative correlation with the progression of circularity in the trachea (P-Cr_Trachea, ρ = 0.66), while showing moderate positive correlations with the progression of wall thickness and angle in the trachea (P-WT_Trachea and P-Angle_Trachea) with ρ values of 0.18 and 0.11, respectively. Similarly, P-FVC displayed a negative correlation with P-Cr_Trachea (ρ = −0.41) and a weaker positive correlation with the progression of normal lung tissue (P-Norm, ρ = 0.18). The progression of FEV_1_/FVC (P-FEV1/FVC) exhibited a positive correlation with P-Angle_Trachea (ρ = 0.31) and was also positively correlated with the progression of emphysema (P-Emph) and consolidation (P-Conso), with ρ values of 0.31 and 0.21, respectively. In contrast, it had strong negative correlations with the progression of normal lung tissue (P-Norm), semi-consolidation (P-Semiconso), and fibrosis (P-Fibr), with ρ values of −0.41, −0.40, and −0.27, respectively. Among the tracheal structural variables, the progression of hydraulic diameter in the trachea (P-Dh_Trachea) showed a positive correlation with P-Conso (ρ = 0.25) and negative correlations with the progression of total blood vessel volume (P-TBV), P-Fibr, P-Norm, and P-Semiconso, with ρ values ranging from −0.29 to −0.23. Additionally, P-WT_Trachea and P-Angle_Trachea showed negative correlations with P-Emph, with ρ values of −0.27 and −0.21, respectively. ADI showed positive correlations with P-FEV1 (ρ = 0.35) and P-WT_Trachea (ρ = 0.21), weak correlations with P-FVC (ρ = 0.06) and P-FEV1/FVC (ρ = 0.04), and negative correlations with P-Cr_Trachea (ρ = −0.36) and P-Semiconso (ρ = −0.20). J exhibited moderate positive correlations with P-FEV1 (ρ = 0.26), P-Conso (ρ = 0.20), P-WT_Trachea (ρ = 0.29), and negative correlations with P-Norm (ρ = −0.20) and P-Cr_Trachea (ρ = −0.42). In contrast, SRI demonstrated positive correlations with P-FEV1 (ρ = 0.38) and P-FVC (ρ = 0.32). SRI also showed a weaker positive correlation with P-Emph (ρ = 0.25) and P-Norm (ρ = 0.23), we well as negative correlations with P-Conso (ρ = −0.37) and P-Angle_Trachea (ρ = −0.22).

## 4 Discussion

This study provides the longitudinal assessment on the structural and functional lung changes in coal workers’ pneumoconiosis using deep learning-based super-resolution and image registration techniques. By enhancing image quality and enabling precise quantification of regional deformations, we have uncovered subtle yet significant airway remodeling, vascular changes, and parenchymal alterations that contribute to disease progression.

The findings of airway remodeling, specifically changes in airway angles, diameters, and circularity, are consistent with previous reports of small airway disease in pneumoconiosis (Fan et al., 2022). However, our study extends these insights by demonstrating that trifurcation angles and hydraulic diameters change predominantly in secondary and tertiary airway branches, which have not been previously characterized in pneumoconiosis (see Supplementary Material for detailed findings). This suggests that localized airway narrowing may precede global airflow obstruction, a phenomenon also observed in chronic obstructive pulmonary disease but not well documented in dust-related lung diseases (McDonough et al., 2011; Singh, 2017). While our study primarily focused on airway-level changes, particularly in secondary and tertiary branches, it is important to acknowledge the potential role of distal structures such as alveolar ducts and sacs in contributing to observed parenchymal alterations. These regions, being the terminal components of the respiratory tree, are highly susceptible to dust deposition and inflammatory injury in pneumoconiosis. Structural remodeling in these areas, although not directly quantified in this study, may underlie the observed reductions in normal lung tissue and vascular volumes, particularly in the upper lobes. Future investigations incorporating high-resolution imaging or histopathological correlation could help elucidate changes in alveolar architecture and their functional consequences.

Additionally, the significant decrease in total blood vessel volume indicates a potential link between pneumoconiosis and vascular remodeling. Changes in pulmonary vasculature, including vasoconstriction and vessel loss, have been implicated in the pathophysiology of chronic lung diseases and may contribute to the development of pulmonary hypertension (Shimoda and Laurie, 2013). The significant reductions in BV11-BV20 in the left upper lung (see Supplementary Material for detailed findings) suggest regional heterogeneity in vascular loss, which may be linked to localized parenchymal destruction or inflammatory processes. This raises important clinical considerations regarding the role of vascular health in the progression of pneumoconiosis and potential targets for therapeutic intervention.

In terms of functional lung metrics, stability in emphysema, ground-glass opacity, and fibrosis percentages suggest that while structural changes are ongoing, considerable functional decline could occur later, reflecting the chronic and progressive nature of pneumoconiosis. However, the notable decrease in normal lung tissues within the right upper lobe could signify early functional decline. This reduction in healthy lung parenchyma could be an early indicator of impaired gas exchange and reduced lung compliance, which are critical factors affecting overall respiratory function (Barrocas et al., 1971; Qureshi, 2011). The stability in emphysema and ground-glass opacity aligns with the understanding that these features represent different pathological processes. Emphysema, characterized by alveolar destruction, and ground-glass opacity, indicative of inflammation and partial filling of air spaces, may not progress uniformly across all lung regions, thereby explaining their relative stability in this study (Mets et al., 2012). Nevertheless, the localized decrease in normal lung tissue underscores the importance of regional assessments in detecting early functional impairments that may not yet be apparent in global lung function tests.

Our image registration-based deformation metrics (ADI, SRI, and J) further emphasize the regional variability of lung deformation over time. Notably, the lower lobes (LLL and RLL) exhibited higher deformation indices (higher median values for ADI and J), indicating significant structural changes and volume expansion compared to the upper lobes. This regional variability in deformation patterns emphasizes the importance of localized analysis rather than global lung function metrics, which may obscure early structural changes. The elevated ADI values suggest a more pronounced directional preference in deformation in these regions, likely reflecting localized mechanical stress or remodeling processes. The SRI, while relatively uniform across the lungs, displayed subtle variations in the RML, highlighting localized directional preferences in deformation. These findings suggest that the lower regions of the lung may be more prone to mechanical stress or disease-related changes over time.

Correlation analysis reveals subtle interactions between functional and structural lung metrics. Although the CT-derived airway metrics showed relatively modest changes over 1 year, they provide detailed structural insights that complement global functional assessments like FEV_1_. While FEV_1_ significantly decreased during follow-up, it does not localize airway changes or distinguish between different remodeling patterns. Our imaging analysis enables quantification of airway wall thickening, luminal narrowing, and lobar-specific alterations, which may precede or accompany functional decline and are valuable for personalized evaluation and disease phenotyping. The potential correlation between the progression of FEV_1_/FVC ratios and both emphysema and consolidation suggests that as airway obstruction increases (reflected by a lower FEV_1_/FVC after a 1-year period), there is a simultaneous increase in emphysematous changes and consolidation. This relationship highlights the interplay between airway obstruction and parenchymal alterations, where structural airway narrowing may exacerbate or be exacerbated by underlying parenchymal damage (Schroeder et al., 2013). In contrast, the negative correlations between FEV_1_/FVC progression and normal lung tissue, semi-consolidation, and fibrosis indicate that as the disease progresses, the loss of healthy lung tissue and the increase in fibrotic changes contribute to the decline in pulmonary function. The inverse relationship between hydraulic diameter progression and fibrosis, as well as blood vessel metrics, suggests that vascular and fibrosis changes may prevent compensatory airway dilation, thereby limiting the lung’s ability to adapt to structural constraints (Hopkins and McLoughlin, 2002). Moreover, the negative correlations observed between tracheal structural variables (angle and wall thickness) and emphysema highlight the complex dynamics of airway and parenchymal interactions. Increased emphysema, characterized by alveolar destruction, could result in altered airway mechanical forces, influencing their structural integrity and function (Paré and Mitzner, 2012). ADI’s positive correlation with the progression of FEV1 and tracheal wall thickness suggests anisotropic deformations may help preserve airflow, while its negative association with tracheal circularity and semi-consolidation highlights sensitivity to structural impairments. J captures volumetric changes tied to airway remodeling (positive with P-FEV1 and P-WT_Trachea) and parenchymal loss (negative with P-Norm and P-Cr_Trachea). SRI’s positive correlation with the progression of FEV1 and FVC suggests compensatory deformation supporting lung function, while its negative association with the progression of consolidation and tracheal angle highlights localized structural compromise. Together, these findings highlight the complementary roles of these metrics in linking structural deformations to functional changes, emphasizing their utility for a more comprehensive understanding of pneumoconiosis progression.

Overall, this study contributes to the literature by identifying specific structural and functional variables that could serve as progression markers for pneumoconiosis. These metrics could enhance early detection strategies, enabling clinicians to identify individuals at higher risk of rapid disease progression and tailor interventions accordingly. Furthermore, the study’s findings on vascular remodeling open avenues for exploring targeted therapies that address both airway and vascular components of pneumoconiosis. The use of a super-resolution model to enhance qCT image quality improved the accuracy of metric extraction, ensuring reliable assessments. Furthermore, beyond enhancing visual clarity, SR reconstruction significantly improved the sensitivity and robustness of quantitative assessments. While Supplementary Section 2.1 shows that SR images enabled comparable or better airway branch identification, even rescuing cases where LR images failed, our comparative analysis in Supplementary Section 2.3 further demonstrated that SR-derived metrics captured additional significant changes not detected in LR images. Additionally, registration between inspiration images at baseline and 1-year follow-up allowed for precise quantification of regional lung deformations, enabling a more detailed understanding of structural changes over time in cases where expiration images were unavailable. However, this study has several limitations. First, the exploration of additional functional variables was constrained by the absence of functional residual capacity scans, which could provide further insights into lung volumes and gas exchange dynamics. Second, the relatively small cohort size limits the generalizability of the findings to the broader pneumoconiosis population and may reduce the statistical power to detect subtle changes. Third, the 1-year interval between diagnoses may be insufficient to capture the full spectrum of disease progression, particularly in a chronic condition like pneumoconiosis, where changes can occur gradually over extended periods. Fourth, although qCT provides valuable structural detail, it involves exposure to ionizing radiation, which can limit the frequency of follow-up assessments. While radiation dose was optimized using Care Dose modulation, patient safety remains a concern, especially in long-term monitoring. Emerging non-invasive techniques such as magnetic resonance elastography (MRE) offer radiation-free alternatives for assessing lung stiffness. Recent studies have validated MRE for evaluating lung function in post-COVID-19 patients and smokers, highlighting its potential for use in pneumoconiosis as well (Bensamoun et al., 2025a; Bensamoun et al., 2025b). Integration of MRE with qCT could enhance tissue characterization while minimizing radiation burden. Finally, patient follow-up remains a major challenge, as it depends on voluntary participation, which can lead to attrition bias and limit the completeness of longitudinal data. It is also important to consider that some structural and functional changes observed in this cohort may be partially influenced by age-related alterations in lung tissue. Aging is associated with reduced lung elasticity, increased airway wall thickness, and changes in vascular compliance, which could confound the interpretation of disease-specific remodeling. While our study primarily attributes these changes to pneumoconiosis, the lack of an age-matched healthy control group limits our ability to fully disentangle aging effects from disease progression. Future research may benefit from a larger cohort with extended follow-up periods to validate these findings and explore additional variables potentially predictive of disease progression, particularly during both inhalation and exhalation phases.

In summary, this longitudinal qCT study elucidates significant structural and vascular alterations in CWP patients, as well as stable yet regionally variable functional metrics. The interplay between airway narrowing, compensatory vascular dilation, and vascular remodeling underscores the complexity of pneumoconiosis progression. These findings support the use of integrated diagnostic methods that combine structural, functional, and vascular assessments to enhance prognostic accuracy and inform targeted interventions. Despite certain limitations, this research advances the understanding of pneumoconiosis pathophysiology and sets the stage for future studies aimed at improving clinical outcomes for affected individuals.

## Data Availability

The original contributions presented in the study are included in the article/Supplementary Material, further inquiries can be directed to the corresponding authors.

## Glossary

1D: One-dimensional
ADI: Anisotropic deformation index
BMI: Body mass index
BV: Blood vessel
CDC: Centers for Disease Control and Prevention
Conso: Consolidation
Cr: Circularity
CT, qCT: Computed tomography, quantitative computed tomography
CWP: Coal workers’ pneumoconiosis
**D**_**inner**_: Average (inner) diameter
**D**_**h**_: Hydraulic diameter
**D**_**outer**_: Outer diameter
Emph: Emphysema
**FEV**_**1**_: Forced expiratory volume in one second
Fibr: Fibrosis
FVC: Forced vital capacity
GGO: Ground-glass opacity
HU: Hounsfield unit(s)
J: Jacobian
LA: Luminal area
LLL: Left lower lobe
LMB: Left main bronchus
LUL: Left upper lobe
MRE: Magnetic resonance elastography
Norm: Normal
P-Angle_Trachea: Progression of angle in trachea
P-Cr_Trachea: Progression of circularity in trachea
P-Dh_Trachea: Progression of hydraulic diameter in trachea
**P**_**e**_: Luminal area perimeter
P-Emph: Progression of emphysema in total lung
P-FEV1/FVC: Progression of FEV_1_/FVC
P-Fibr: Progression of fibrosis in total lung
PFT: Pulmonary functional test
P-Norm: Progression of normal lung tissue in total lung
P-Semiconso: Progression of semi-consolidation in total lung
P-TBV: Progression of total blood vessel
P-WT_Trachea: Progression of wall thickness in trachea
RLL: Right lower lobe
RMB: Right main bronchus
RML: Right middle lobe
RUL: Right upper lobe
Semiconso: Semi-consolidation
sLLL: Subgroup of the left lower lobe
sLUL: Subgroup of the left upper lobe
SRI: Slab rod index
sRLL: Subgroup of the right lower lobe
sRML: Subgroup of the right middle lobe
sRUL: Subgroup of the right upper lobe
TBV: Total blood volume
TLC: Total lung capacity
TriLLB: Trifurcation of the left lower lobe
TriLUL: Trifurcation of the left upper lobe
TriRLL: Trifurcation of the right lower lobe
TriRUL: Trifurcation of the right upper lobe
WL: Whole lung
WT: Wall thickness
θ: Bifurcation angle

## References

[B1] Akira M. Inoue Y. Kitaichi M. Yamamoto S. Arai T. Toyokawa K. (2009). Usual interstitial pneumonia and nonspecific interstitial pneumonia with and without concurrent emphysema: thin-section CT findings. Radiology 251 (1), 271–279. 10.1148/radiol.2511080917 19221055

[B2] Avants B. B. Tustison N. J. Stauffer M. Song G. Wu B. Gee J. C. (2014). The Insight ToolKit image registration framework. Front. neuroinformatics 8, 44. 10.3389/fninf.2014.00044 PMC4009425 24817849

[B3] Barrocas M. Nuchprayoon C. V. Claudio M. King F. W. Danon J. Sharp J. T. (1971). Gas exchange abnormalities in diffuse lung disease. Am. Rev. Respir. Dis. 104 (1), 72–87. 10.1164/arrd.1971.104.1.72 5556233

[B4] Bensamoun S. F. McGee K. P. Chakouch M. Pouletaut P. Charleux F. (2025a). Monitoring of lung stiffness for long-COVID patients using magnetic resonance elastography (MRE). Magn. Reson. Imaging 115, 110269. 10.1016/j.mri.2024.110269 39491570

[B5] Bensamoun S. F. McGee K. P. Chakouch M. Pouletaut P. Charleux F. (2025b). Quantification of lung stiffness using magnetic resonance elastography (MRE): clinical validation for smokers. IEEE Trans. Biomed. Eng., 1–8. 10.1109/TBME.2025.3553375 40111770

[B6] Blackley D. J. Halldin C. N. Laney A. S. (2018). Continued increase in prevalence of coal workers' pneumoconiosis in the United States, 1970-2017. Am. J. Public Health 108 (9), 1220–1222. 10.2105/AJPH.2018.304517 30024799 PMC6085042

[B7] Blanc P. D. Seaton A. (2016). Pneumoconiosis Redux. Coal Workers’ pneumoconiosis and silicosis are still a problem. American Thoracic Society, 603–605. 10.1164/rccm.201511-2154ED 26977968

[B8] Castranova V. Vallyathan V. (2000). Silicosis and coal workers' pneumoconiosis. Environ. health Perspect. 108 (Suppl. 4), 675–684. 10.1289/ehp.00108s4675 PMC1637684 10931786

[B9] Chae K. J. Jin G. Y. Choi J. Lee C. H. Choi S. Choi H. (2021). Generation-based study of airway remodeling in smokers with normal-looking CT with normalization to control inter-subject variability. Eur. J. radiology 138, 109657. 10.1016/j.ejrad.2021.109657 33773402

[B10] Chau N.-K. Ma T.-T. Kim W. J. Lee C. H. Jin G. Y. Chae K. J. (2024). BranchLabelNet: anatomical human airway labeling approach using a dividing-and-grouping multi-label classification. Med. and Biol. Eng. and Comput. 62, 3107–3122. 10.1007/s11517-024-03119-7 38777935

[B11] Chen Y. H. Shi F. Christodoulou A. G. Xie Y. B. Zhou Z. W. Li D. B. (2018). Efficient and accurate MRI super-resolution using a generative adversarial network and 3D multi-level densely connected network. Medical image computing and computer assisted intervention - miccai 2018. 11070:91–99.

[B12] Choi S. Hoffman E. A. Wenzel S. E. Castro M. Fain S. B. Jarjour N. N. (2015). Quantitative assessment of multiscale structural and functional alterations in asthmatic populations. J. Appl. physiology 118 (10), 1286–1298. 10.1152/japplphysiol.01094.2014 PMC4436982 25814641

[B13] Choi S. Hoffman E. A. Wenzel S. E. Tawhai M. H. Yin Y. B. Castro M. (2013). Registration-based assessment of regional lung function via volumetric CT images of normal subjects vs. severe asthmatics. J. Appl. Physiology 115 (5), 730–742. 10.1152/japplphysiol.00113.2013 PMC3763069 23743399

[B14] Colombi D. Bodini F. C. Petrini M. Maffi G. Morelli N. Milanese G. (2020). Well-aerated lung on admitting chest CT to predict adverse outcome in COVID-19 pneumonia. Radiology 296 (2), E86–E96. 10.1148/radiol.2020201433 32301647 PMC7233411

[B15] Cullinan P. Reid P. (2013). Pneumoconiosis. Journal 22 (2), 249–252. 10.4104/pcrj.2013.00055 PMC6442808 23708110

[B16] Fan Y. Ma R. Du X. Chai D. Yang S. Ye Q. (2022). Small airway dysfunction in pneumoconiosis: a cross-sectional study. BMC Pulm. Med. 22 (1), 167. 10.1186/s12890-022-01929-9 35484546 PMC9052448

[B17] Hopkins N. McLoughlin P. (2002). The structural basis of pulmonary hypertension in chronic lung disease: remodelling, rarefaction or angiogenesis? J. Anat. 201 (4), 335–348. 10.1046/j.1469-7580.2002.00096.x 12430958 PMC1570922

[B18] Hoy R. F. Chambers D. C. (2020). Silica‐related diseases in the modern world. Allergy 75 (11), 2805–2817. 10.1111/all.14202 31989662

[B19] Kim T. Lim M.-n. Kim W. J. Ho T. T. Lee C. H. Chae K. J. (2022). Structural and functional alterations of subjects with cement dust exposure: a longitudinal quantitative computed tomography-based study. Sci. Total Environ. 837, 155812. 10.1016/j.scitotenv.2022.155812 35550893

[B20] Liu F. Zhang Q. Huang C. Shi C. Wang L. Shi N. (2020). CT quantification of pneumonia lesions in early days predicts progression to severe illness in a cohort of COVID-19 patients. Theranostics 10 (12), 5613–5622. 10.7150/thno.45985 32373235 PMC7196293

[B21] Matsuoka S. Yamashiro T. Matsushita S. Kotoku A. Fujikawa A. Yagihashi K. (2015). Quantitative CT evaluation in patients with combined pulmonary fibrosis and emphysema: correlation with pulmonary function. Acad. Radiol. 22 (5), 626–631. 10.1016/j.acra.2015.01.008 25728361

[B22] McDonough J. E. Yuan R. Suzuki M. Seyednejad N. Elliott W. M. Sanchez P. G. (2011). Small-airway obstruction and emphysema in chronic obstructive pulmonary disease. N. Engl. J. Med. 365 (17), 1567–1575. 10.1056/NEJMoa1106955 22029978 PMC3238466

[B23] Mets O. De Jong P. Van Ginneken B. Gietema H. Lammers J. (2012). Quantitative computed tomography in COPD: possibilities and limitations. Lung 190, 133–145. 10.1007/s00408-011-9353-9 22179694 PMC3310986

[B24] Momtazmanesh S. Moghaddam S. S. Ghamari S.-H. Rad E. M. Rezaei N. Shobeiri P. (2023). Global burden of chronic respiratory diseases and risk factors, 1990–2019: an update from the Global Burden of Disease Study 2019. EClinicalMedicine 59, 101936. 10.1016/j.eclinm.2023.101936 37229504 PMC7614570

[B25] Paré P. D. Mitzner W. (2012). Airway-parenchymal interdependence. Compr. Physiol. 2 (3), 1921–1935. 10.1002/cphy.c110039 23723029 PMC4557883

[B26] Pyo J. Chau N.-K. Park E.-K. Choi S. (2023). Computed tomography-based imaging biomarker identifies coal workers’ pneumoconiosis. Front. Physiology 14, 1288246. 10.3389/fphys.2023.1288246 PMC10702505 38074321

[B27] Qi X.-M. Luo Y. Song M.-Y. Liu Y. Shu T. Pang J.-L. (2021). Pneumoconiosis: current status and future prospects. Chin. Med. J. 134 (08), 898–907. 10.1097/CM9.0000000000001461 33879753 PMC8078400

[B28] Qureshi S. M. (2011). Measurement of respiratory function: an update on gas exchange. Anaesth. and Intensive Care Med. 12 (11), 490–495. 10.1016/j.mpaic.2011.08.006

[B29] Rabiei P. Ferguson E. C. Hanna M. F. Odisio E. G. Estrada-Y-Martin R. M. Ocazionez D. (2020). Imaging in occupational and environmental lung disease. Curr. Pulmonol. Rep. 9 (3), 63–73. 10.1007/s13665-020-00250-2

[B30] Rosner B. (1982). A generalization of the paired t‐test. J. R. Stat. Soc. Ser. C Appl. Statistics 31 (1), 9–13. 10.2307/2347069

[B31] Rueckert D. Sonoda L. I. Hayes C. Hill D. L. Leach M. O. Hawkes D. J. (1999). Nonrigid registration using free-form deformations: application to breast MR images. IEEE Trans. Med. imaging 18 (8), 712–721. 10.1109/42.796284 10534053

[B32] Schroeder J. D. McKenzie A. S. Zach J. A. Wilson C. G. Curran-Everett D. Stinson D. S. (2013). Relationships between airflow obstruction and quantitative CT measurements of emphysema, air trapping, and airways in subjects with and without chronic obstructive pulmonary disease. Am. J. Roentgenol. 201 (3), W460–W470. 10.2214/AJR.12.10102 23971478 PMC4067052

[B33] Shapiro S. S. Francia R. (1972). An approximate analysis of variance test for normality. J. Am. Stat. Assoc. 67 (337), 215–216. 10.2307/2284728

[B34] Shimoda L. A. Laurie S. S. (2013). Vascular remodeling in pulmonary hypertension. J. Mol. Med. 91, 297–309. 10.1007/s00109-013-0998-0 23334338 PMC3584237

[B35] Singh D. (2017). Small airway disease in patients with chronic obstructive pulmonary disease. Tuberc. Respir. Dis. Seoul. 80 (4), 317–324. 10.4046/trd.2017.0080 28905527 PMC5617847

[B36] Soft C. (2024). AVIEW COPD. Available online at: https://www.corelinesoft.com/en/solutions/copd (Accessed August 20, 2024).

[B37] Soydan L. (2021). The role of high resolution computed tomography in the evaluation of pneumoconiosis. Haydarpaşa Numune Hast. tıp dergisi = Med. J. Haydarpaşa Numune Hosp. 61 (2), 234. 10.14744/hnhj.2020.23590

[B38] Wilcoxon F. (1992). “Individual comparisons by ranking methods,” in Breakthroughs in statistics: methodology and distribution. Springer, 196–202.

[B39] Wissler C. (1905). The Spearman correlation formula. Science 22 (558), 309–311. 10.1126/science.22.558.309 17836577

[B40] Xia L. Lü F. Wang Y. Sheng B. Zhou S. (2012). Compute tomography-based quantitative evaluation of pneumoconiosis. Nan Fang yi ke da xue xue bao= J. South. Med. Univ. 32 (12), 1768–1772. 23268407

