# Supplementary material for: Longitudinal analysis of coal workers’ pneumoconiosis using enhanced resolution-computed tomography images: unveiling patterns in lung structure, function, and clinical correlations
Source: Front Physiol. 2025 May 30;16:1578058. doi: 10.3389/fphys.2025.1578058 (PMC12162278; doi:10.3389/fphys.2025.1578058)

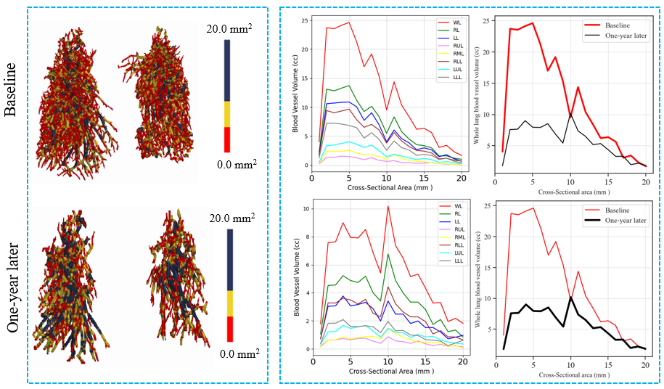

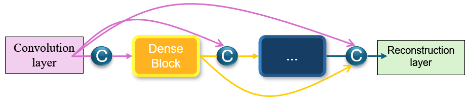

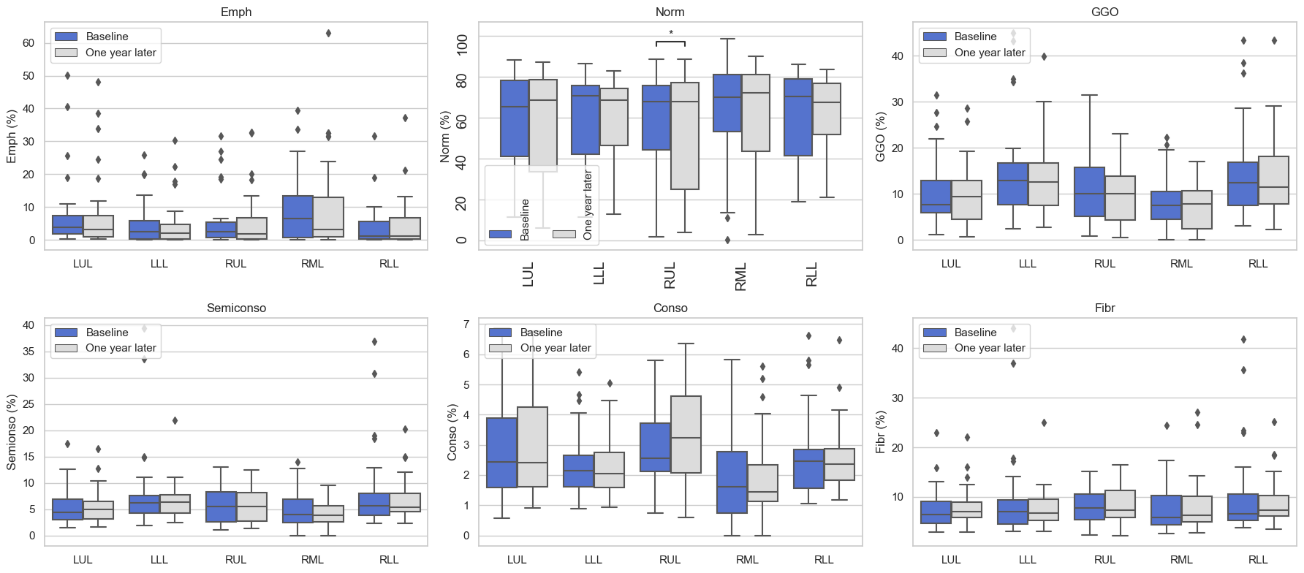

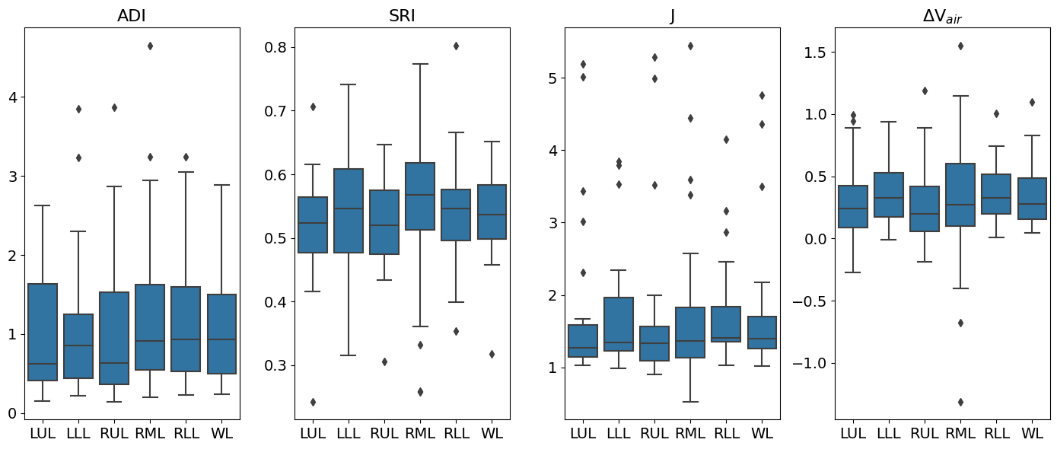

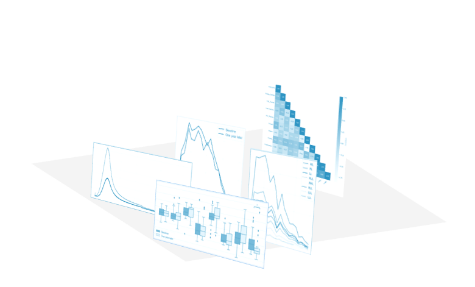

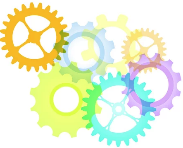

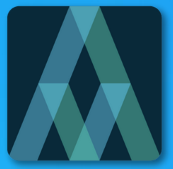

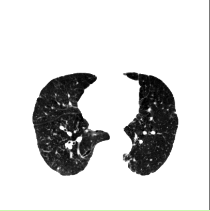

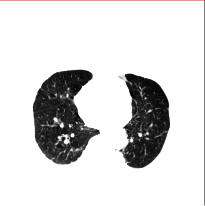

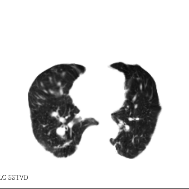

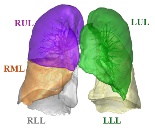

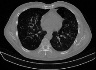

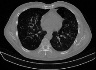

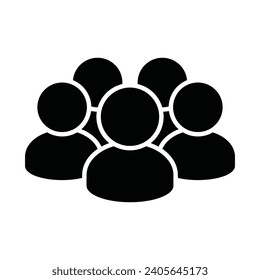


Findings and Conclusion

- Significant airway remodeling in small branches; main airways remained stable.
- Reduced normal lung tissue in the right upper lobe; vascular volume showed significant loss.
- Lower lobes exhibited pronounced deformation and regional volume contraction.
- ADI and SRI effectively captured structural changes and correlated with functional metrics.
- qCT imaging and super-resolution enhancement enable robust pneumoconiosis monitoring.

Study Design

**Reconstructed images**

**Original images**

**Super-resolution model**

Baseline

One-year
follow-up

One-year
follow-up

Baseline

**Registered image**

**Labeled
airway tree**

**Segmented airway tree**

**Lung lobes**

**AVIEW coreline**

Statistical analysis

Feature extraction

Structural and parenchymal functional variables

Pulmonary functional test

Baseline vs. one-year follow-up

**Registration**


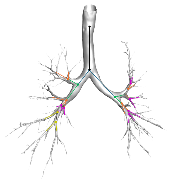

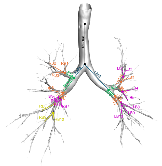


**Branch
Label
Net**

Pre-processing


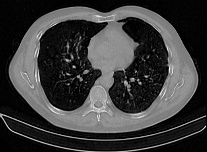

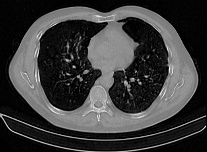

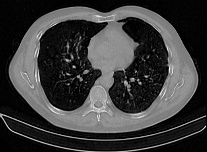

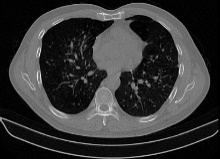

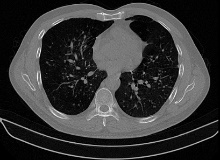

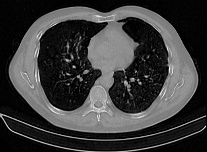

Supplement: Supplementary file 1 [file Supplementaryfile1.docx]
