# Supplementary material for: Longitudinal analysis of coal workers’ pneumoconiosis using enhanced resolution-computed tomography images: unveiling patterns in lung structure, function, and clinical correlations
Source: Front Physiol. 2025 May 30;16:1578058. doi: 10.3389/fphys.2025.1578058 (PMC12162278; doi:10.3389/fphys.2025.1578058)
Supplement: Supplementary file 2 [file Supplementaryfile2.docx]

**Highlights for the study**

- Enhanced resolution of inspiratory qCT images using a super-resolution deep-learning model.

- Identified structural remodeling in small airways and localized vascular volume loss.

- Non-rigid image registration quantified anisotropic deformation and regional volume changes.

- Longitudinal correlations reveal interplay between airway geometry and pulmonary function.

- Findings highlight qCT's potential for monitoring progression in pneumoconiosis patients.
