# Supplementary material for: Longitudinal analysis of coal workers’ pneumoconiosis using enhanced resolution-computed tomography images: unveiling patterns in lung structure, function, and clinical correlations
Source: Front Physiol. 2025 May 30;16:1578058. doi: 10.3389/fphys.2025.1578058 (PMC12162278; doi:10.3389/fphys.2025.1578058)
Supplement: Supplementary file 3 [file DataSheet1.docx]

Supplementary Material

# Adjustments and results of the super-resolution model

## Model overview

We adopted the mDCSRN-GAN (1), a lightweight and effective framework for improving image resolution. This framework uses a multi-level densely connected structure with a deep DenseBlock split into shallow blocks for producing super-resolution (SR) images. Additionally, it uses 1 × 1 × 1 convolutional layers as compressors to reduce memory usage and ensure equal weighting. The discriminator in this framework is similar to than in SRGAN (2), but it uses LayerNorm instead of BatchNorm to distinguish between manufactured and actual data (see Supplementary Figure 1, Discriminator). Moreover, the Wasserstein generative adversarial network with gradient penalty (WGAN-GP) is utilized to stabilize the adversarial training process.

The original mDCSRN-GAN. which utilizes four DenseBlocks, each containing four units, is used to generate a SR image from a 3D LR brain image (1). To adapt this architecture for 3D lung CT imaging, we implemented several modifications through a rigorous fine-tuning process. The optimal configuration was determined to include a margin of eight and five DenseBlocks, each comprising six units (see Supplementary Figure 1, Generator). All other architectural components were maintained based on the original mDCSRN-GAN design (1). These adaptations were the outcome of an extensive parameter optimization study. The margin parameter was evaluated across a range of 1 to 10, while the number of DenseBlocks varied from 4 to 8. Additionally, the number of units within each DenseBlock was assessed at values of 4, 5, and 6. This systematic exploration of the parameter space was conducted to optimize the model's performance specifically for our lung CT dataset. The fine-tuning process was instrumental in tailoring the network architecture to suit the unique characteristics of 3D lung CT images. By methodically exploring these parameter ranges, we were able to identify the configuration that yielded superior performance metrics for our specific application while maintaining the fundamental strengths of the original architecture.

The training process employed in this study strictly followed to the protocol established by Chen et al. (1). We replicated their approach, adhering closely to the original training parameters and procedures. This choice was made to ensure comparability and to build upon the established foundation in the field.

## Experimental results

The dataset we used to train the mDCSRN-GAN model consists of 1,793 high-resolution (HR) lung CT images from a cohort of 899 participants. The images span both the inhalation and exhalation phases and include healthy participants, cement dust-exposed patients, and asthma patients (3-5). The images have a matrix size of 512 × 512 × 320, providing detailed structures ideal for SR model training. The HR images served as the original, ground truth versions. They were then intentionally reduced in quality to create corresponding low-resolution (LR) versions. The data was divided into training, validation, and testing sets with an 8:1:1 split.

The results of the SR model are presented in Supplementary Table 1, and a demonstration of a random subject is shown in Supplementary Figure 2. The relationship between model complexity and performance is illustrated through the correlation of increasing numbers of DenseBlocks and Units (#DenseBlock and #Unit) with the total number of model parameters (#param). The experimental results, obtained after 1,500,000 training steps, indicate that the configuration with five DenseBlocks and six units per block provides an optimal balance between image quality and computational efficiency. While the highest structural similarity index measure (SSIM) (0.8954 ± 0.0394) is achieved by the eight DenseBlocks and six units per block configuration, it requires 1,623,680 parameters. In contrast, the selected configuration achieves a nearly comparable SSIM of 0.8945 (±0.0445) with substantially fewer parameters (984,032), making it a more efficient choice. It also achieves the low normalized root mean square error (NRMSE) (0.1479 ± 0.1078) among all configurations and a competitive peak signal-to-noise ratio (PSNR) (33.3974 ± 5.0605), close to the highest observed. Although the five DenseBlocks and four units per block model yields the highest PSNR (33.5194 ± 5.1393), it comes with a significantly lower SSIM (0.8809 ± 0.4091), indicating less structural fidelity. These results support our selection of the five DenseBlocks and six units per block configuration as offering the most favorable trade-off between perceptual quality and model complexity for super-resolution reconstruction of lung CT images.

# Longitudinal analysis of coal workers’ pneumoconiosis

## Enhancement of CT images for improved pneumoconiosis detection

The cohort of coal workers with pneumoconiosis consisted of 62 original low-resolution CT scans taken at baseline and during a one-year follow-up. Two of these scans exhibited significant degradation in image quality, preventing processing by AVIEW software (6). The trained mDCSRN-GAN model was employed to generate higher resolution versions of the original images.

Supplementary Figure 3 demonstrates the efficacy of our adapted method by providing a comparative visualization of original and enhanced images from a randomly selected case within our cohort.

The reconstructed SR images offer improved visualization that can potentially enhance diagnostic accuracy for clinicians. Furthermore, they contribute to statistical analysis by enabling the identification of a comparable number of airway branches to the original LR images, as detailed in Supplementary Table 2.

Supplementary Table 2 presents a comparison of the airway branch counts obtained from both LR and SR images at baseline and at the one-year follow-up. At baseline, the mean number of airway branches identified in LR images was 31.26 (±5.64) across 31 subjects, while the SR images yielded a mean of 31.97 (±5.57) branches for the same cohort. At the one-year follow-up, the mean branch count in LR images was 29.77 (±9.55) in 29 subjects (processing of two LR images was unsuccessful), and the SR images showed a mean of 31.45 (±5.51) in all 31 subjects. These results indicate that while the SR reconstruction did not lead to a substantial increase in the total number of main airway branches identified compared to the LR images, it did allow for successful analysis in cases where LR image quality was degraded (as seen in the one-year follow-up). The comparable branch counts suggest that the SR method preserves the overall structural information relevant for identifying main airway branches while potentially offering improved visualization and robustness to image quality issues.

## Additional statistical analysis results

Supplementary Table 3 illustrates the changes in structural and quantitative variables observed in the cohort of coal workers’ pneumoconiosis subjects (N = 31) between baseline and one-year follow-up. The statistical analysis highlights significant structural changes across multiple variables. Notable alterations in angle variables were observed in both the left and right bronchial branches, including LB4+5, LB9+10, LB3, LB4, LB5, RB9+10, RB2, RB3, RB4, RB5, RB6, RB10, and the trifurcation angle of the right lower lobe (TriRLL). In addition, significant changes in hydraulic diameter were noted at LB1+2, LB2, LB3, and the trifurcation of the left lower lobe (TriLLB), with p-values ranging from 0.011 to 0.03. Wall thickness analysis revealed contrasting patterns, with an increase in LB1+2+3 (p-value = 0.028) and decreases in RB8 and RB9 (p-values 0.001 and 0.021, respectively). As illustrated in Supplementary Figure 4, the wall thickness in patients generally decreased after one year. Lastly, circularity measurements indicated significant changes at RB2 (p = 0.029), RB4 (p = 0.048), and RB8 (p = 0.0007).

## Comparative analysis of metrics extracted from LR and SR images

To assess the added value of SR reconstruction in quantitative airway analysis, we compared structural, functional, and vascular metrics extracted from both original LR CT images and their SR-enhanced counterparts. The results, summarized in Supplementary Tables 5-7, demonstrate that SR images not only retain key significant findings observed in LR images but also reveal additional changes, particularly in hydraulic diameter and functional parameters. These findings highlight the potential of SR reconstruction to improve the sensitivity and anatomical completeness of image-derived metrics in longitudinal studies.

# Supplementary figures and tables

## Supplementary figures


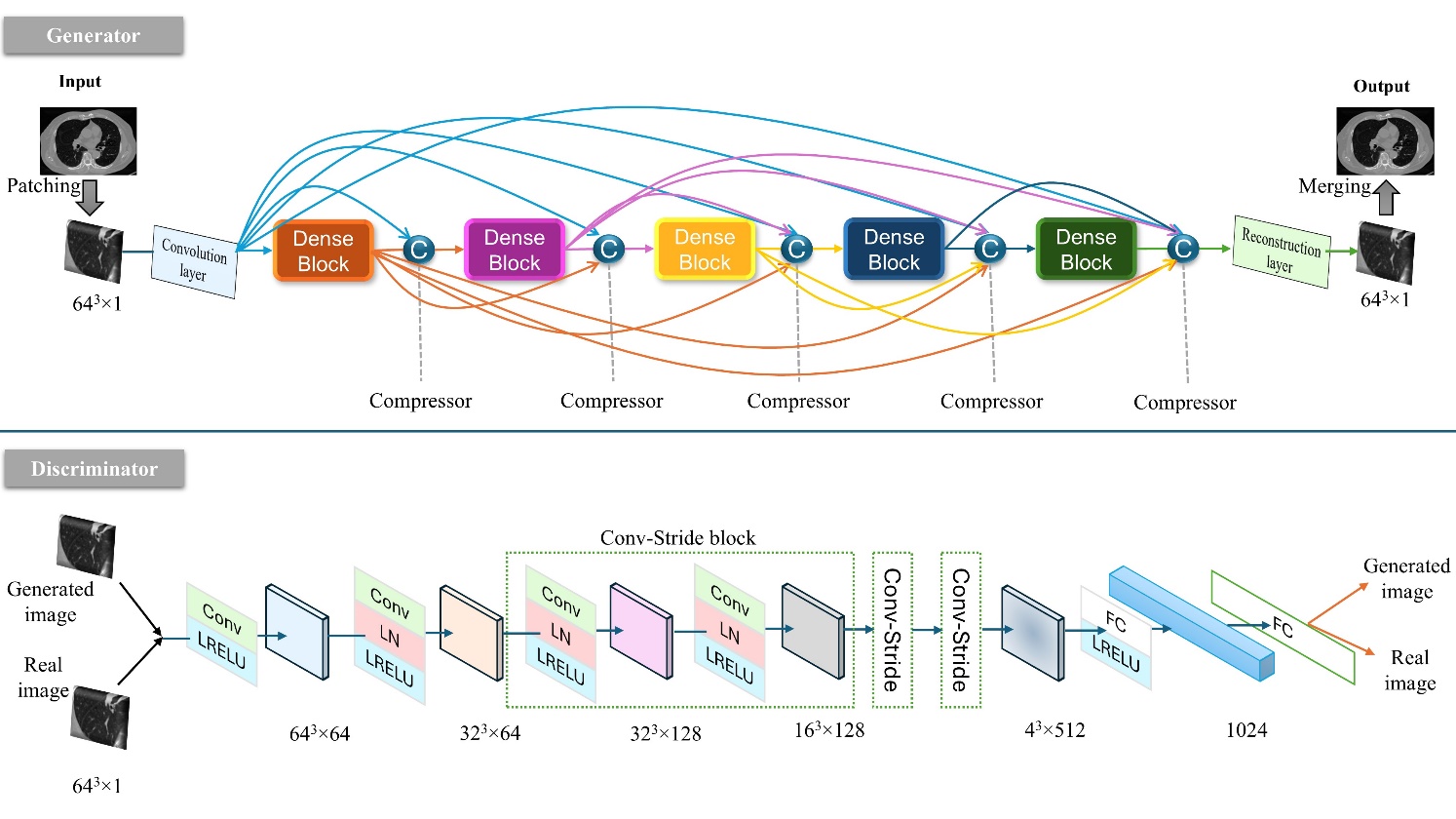


Supplementary Figure 1. Architecture of 3D CT lung super-resolution model. The generator has five DenseBlocks, with each DenseBlock containing six units. The discriminator utilized in this model is based on the study conducted by Chen et al. (1). Conv, convolutional; LRELU, leaky RELU; LN, LayerNorm; FC, fully connected.

| 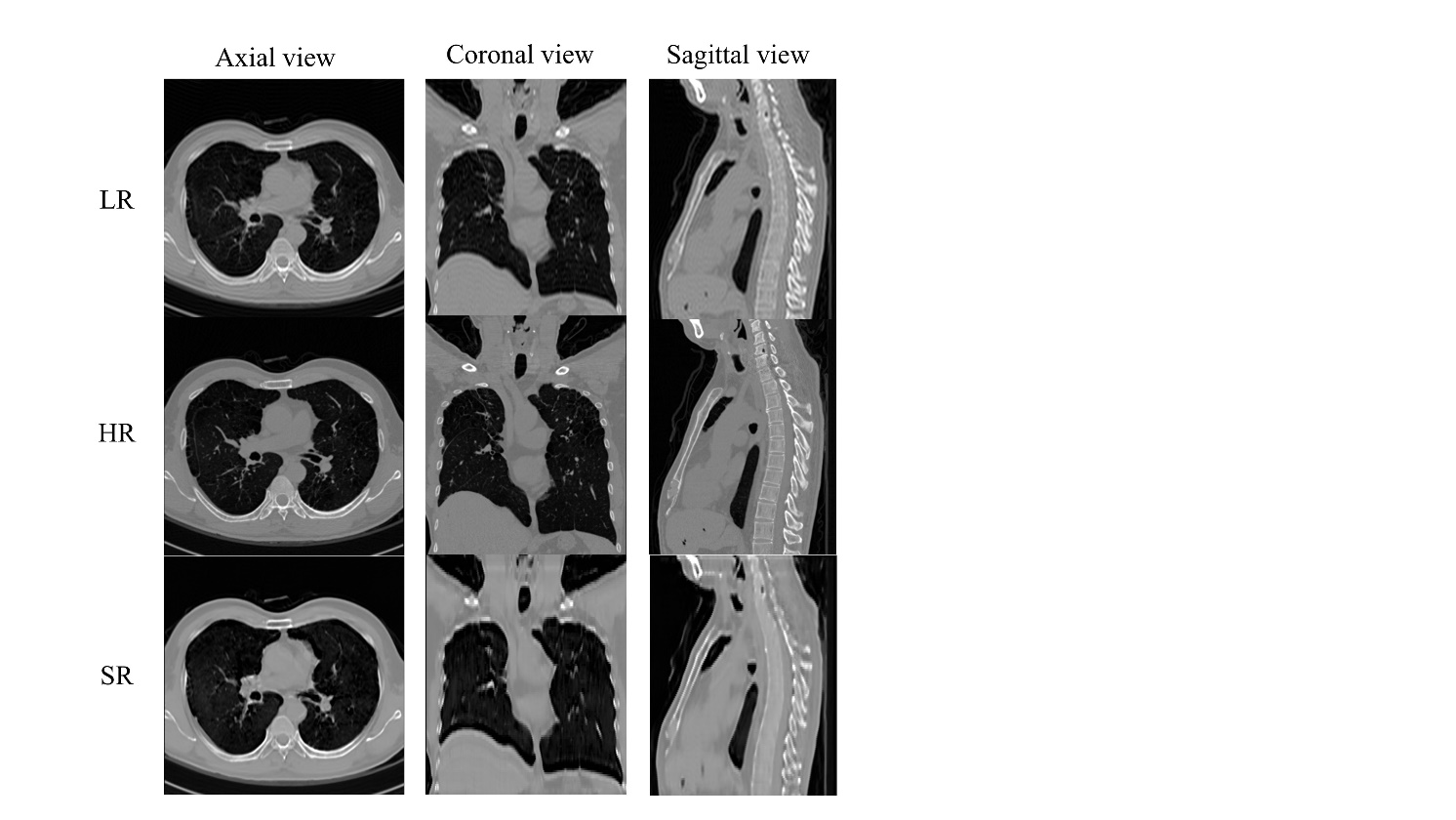  **(A)** |
| --- |
| 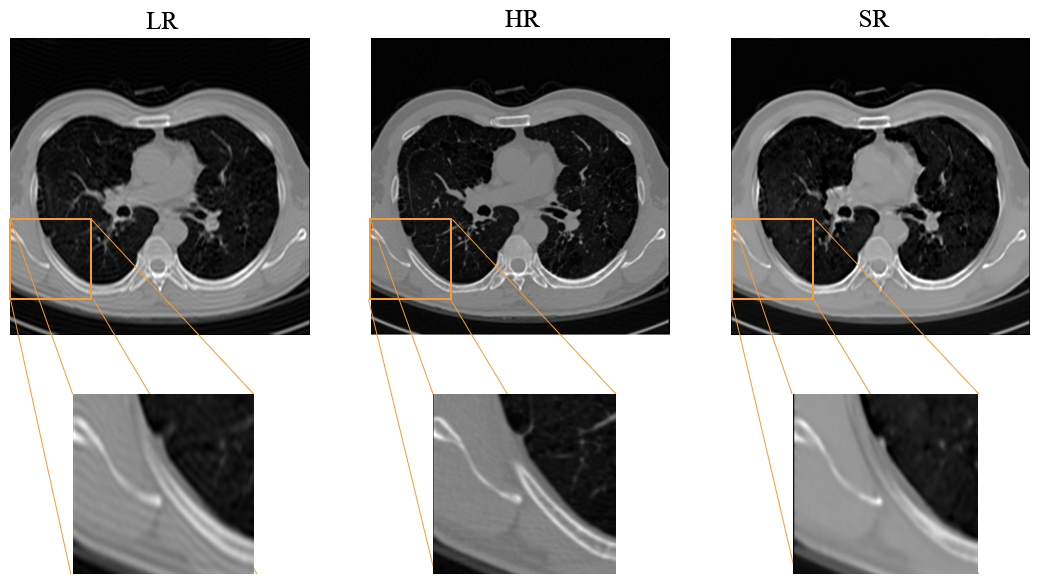  **(B)** |
| 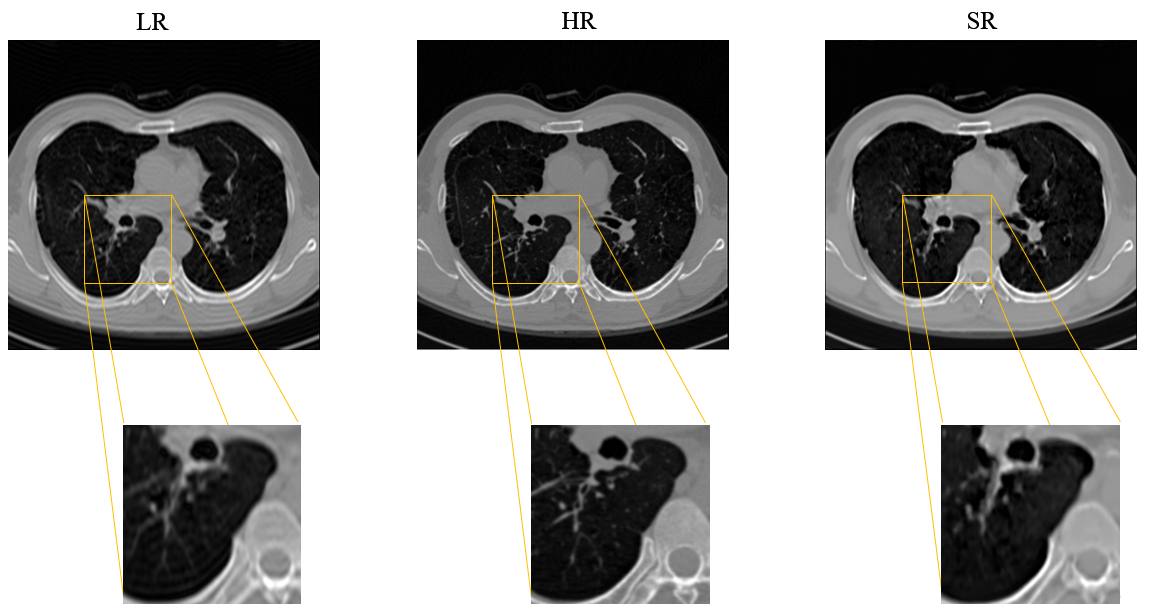  **(C)** |

Supplementary Figure 2. Illustration of SR reconstruction results. (A) shows axial, coronal, and sagittal views. (B) and (C) provide a magnified view of selected axial regions to highlight differences in structural clarity and edge definition. The SR reconstruction more closely approximates HR quality than LR, particularly in anatomical boundaries and fine textures.

| **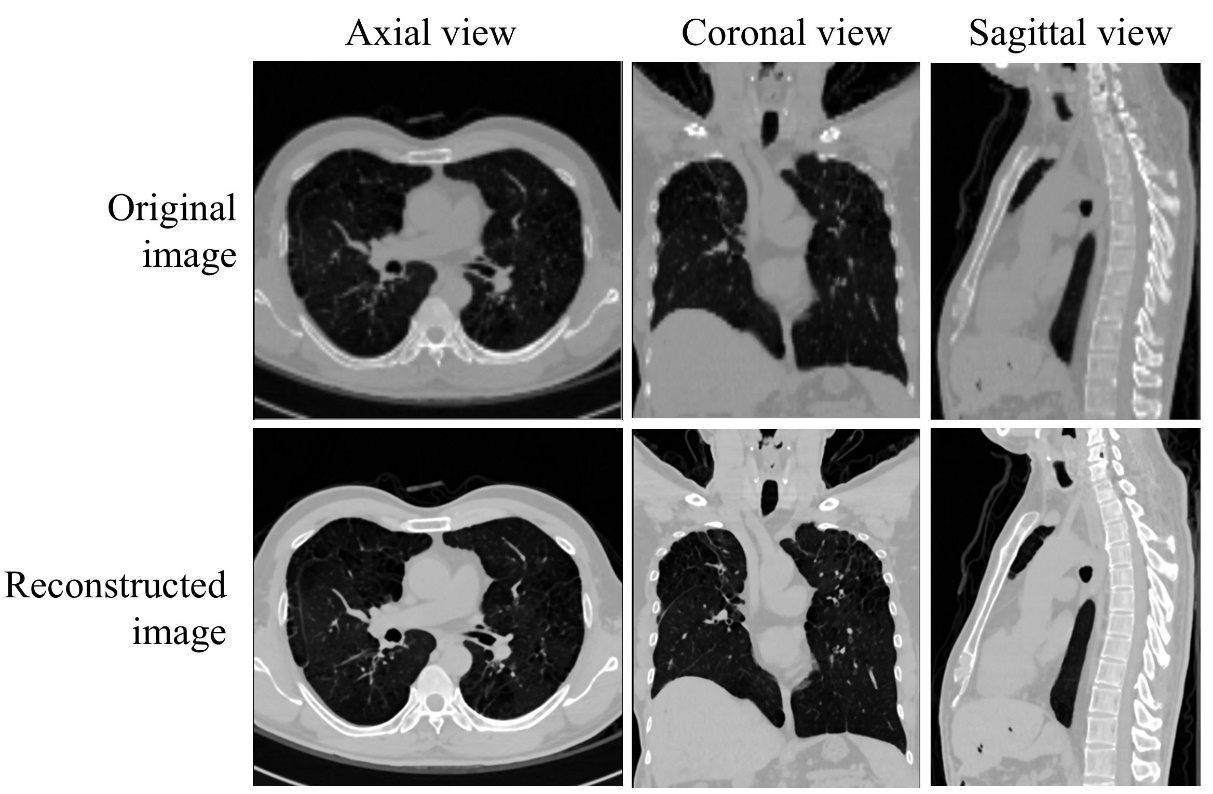**  **(A)** |
| --- |
| **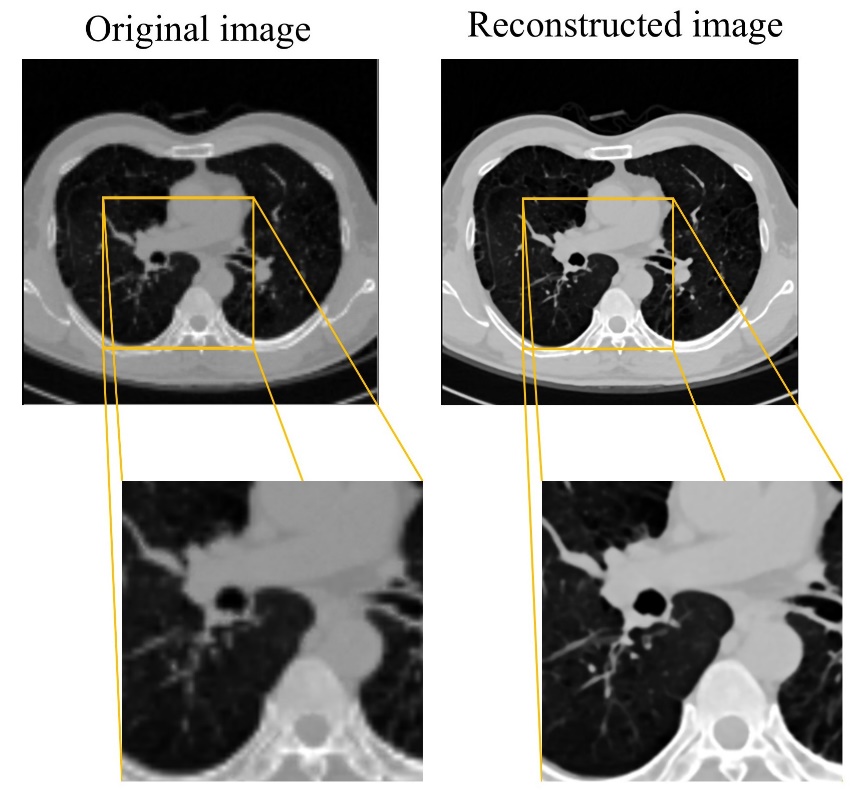**  **(B)** |

**Supplementary Figure 3.** An example of a generated image of a randomly selected coal worker with pneumoconiosis subject. (A) A comparison of the original image and the reconstructed image from different perspectives, including axial, coronal, and sagittal planes. (B) A magnified view of selected regions from (A), highlighting the detailed differences between the original and reconstructed images. The reconstructed image demonstrates improved sharpness of airway structures and enhanced contrast, facilitating better visual delineation of pulmonary features.


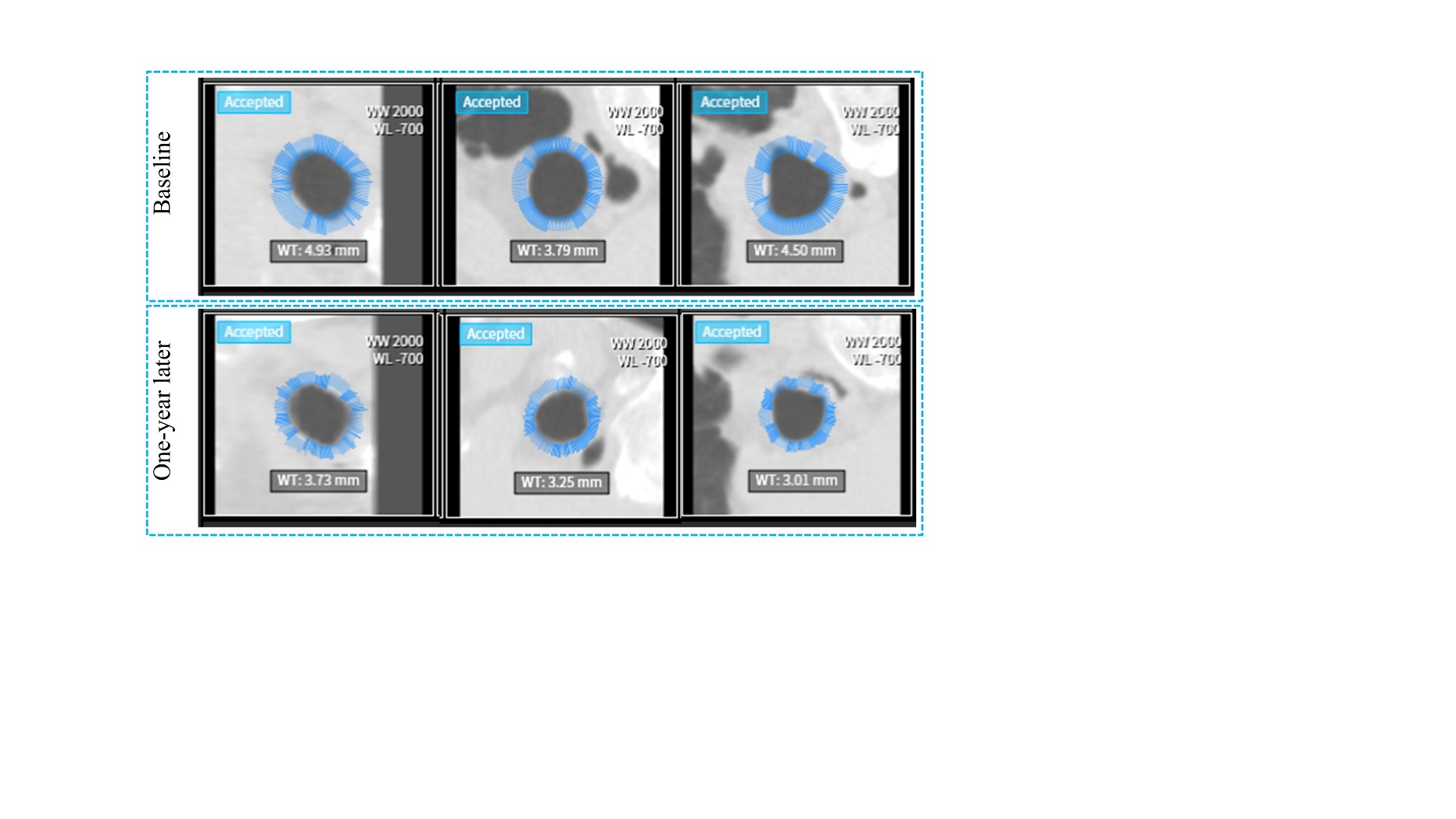


Supplementary Figure 4. Assessment of tracheal wall thickness (WT) in a representative subject at baseline and at the one-year follow-up.

## Supplementary tables

Supplementary Table 1. Quantitative image quality assessment across various SR model configurations. SSIM, structural similarity index measure; PSNR, peak signal-to-noise ratio; NRMSE, normalized root mean square error.

| **#DenseBlock** | **#Unit** | **#param** | **SSIM↑** | **PSNR↑** | **NRMSE↓** |
| --- | --- | --- | --- | --- | --- |
| 4 | 4 | 411,648 | 0.7858 (0.0429) | 32.5814 (4.0974) | 0.1527 (0.0933) |
|  | 5 | 581,440 | 0.8906 (0.0455) | 33.2432 (5.3199) | 0.1528 (0.1127) |
|  | 6 | 779,008 | 0.8897 (0.0454) | 33.0141 (5.0104) | 0.1521 (0.1096) |
| **5** | 4 | 522,272 | 0.8809 (0.4091) | 33.5194 (5.1393) | 0.1468 (0.1089) |
|  | 5 | 735,792 | 0.8293 (0.0550) | 23.6284 (4.2196) | 0.4133 (0.1463) |
|  | **6** | **984,032** | **0.8945 (0.0445)** | **33.3974 (5.0605)** | **0.1479 (0.1078)** |
| 6 | 4 | 635,968 | 0.8773 (0.0377) | 33.2569 (4.9617) | 0.1491 (0.1059) |
|  | 5 | 893,728 | 0.8919 (0.0443) | 32.7382 (5.1583) | 0.1628 (0.1241) |
|  | 6 | 1,193,152 | 0.8871 (0.0357) | 32.4994 (4.8880) | 0.1642 (0.1201) |
| 7 | 4 | 752,736 | 0.8951 (0.0403) | 32.8068 (4.5041) | 0.1548 (0.1085) |
|  | 5 | 1,055,248 | 0.8908 (0.0434) | 32.1859 (3.5346) | 0.1548 (0.0839) |
|  | 6 | 1,406,368 | 0.8937 (0.0363) | 30.4980 (3.5627) | 0.1871 (0.0781) |
| 8 | 4 | 872,576 | 0.8925 (0.0308) | 32.2633 (4.0198) | 0.1614 (0.0910) |
|  | 5 | 1,220,352 | 0.8914 (0.0450) | 32.3619 (4.5204) | 0.1630 (0.1137) |
|  | 6 | 1,623,680 | 0.8954 (0.0394) | 32.6283 (3.8678) | 0.1499 (0.0894) |

Supplementary Table 2. Comparison of airway branch counts obtained from LR and SR images at baseline and one-year later. Values are displayed as means with standard deviations (SD) provided in parentheses.

| **Imaging method** | **Time point** | **Mean (SD)** | **Number of subjects** |
| --- | --- | --- | --- |
| LR | Baseline | 31.26 (5.64) | 31 |
|  | One-year later | 29.77 (9.55) | 29^*^ |
| SR | Baseline | 31.97 (5.57) | 31 |
|  | One-year later | 31.45 (5.51) | 31 |
| ^*^Processing of two LR images from the one-year follow-up was unsuccessful due to substantial image quality degradation. | | | |

Supplementary Table 3. The qCT-based airway structural measurements were obtained from baseline and one-year follow-up images. θ, bifurcation angle; D_h_, hydraulic diameter; WT, wall thickness; Cr, circularity; LMB, left main bronchus; RMB, right main bronchus; TriLLB, trifurcation of the left lower lobe; TriLUL, trifurcation of the left upper lobe; TriRLL, trifurcation of the right lower lobe; TriRUL, trifurcation of the right upper lobe; sRUL, subgroup of the right upper lobe; sRML, subgroup of the right middle lobe; sRLL, subgroup of the right lower lobe; sLUL, subgroup of the left upper lobe; sLLL, subgroup of the left lower lobe. Values are displayed as means with standard deviations provided in parentheses.

| **Region** | **Baseline** | **One-year later** | **p-value** | **Q-value** |
| --- | --- | --- | --- | --- |
| **Angle, θ** | | | |  |
| Trachea | 84.462 (13.46) | 83.321 (11.64) | 0.455 | 0.715 |
| LMB | 86.358 (15.4) | 88.635 (20.44) | 0.378 | 0.394 |
| RMB | 101.386 (17.12) | 104.149 (17.68) | 0.622 | 0.754 |
| Bronint | 64.949 (25.85) | 62.284 (20.37) | 0.794 | 0.944 |
| TriLUL | 106.733 (26.57) | 107.403 (24.35) | 0.492 | 0.522 |
| TriLLB | 58.246 (20) | 56.963 (20.4) | 0.742 | 0.834 |
| TriRUL | 72.159 (24.5) | 80.055 (28.15) | 0.14 | 0.272 |
| **TriRLL** | **67.555 (27.47)** | **54.54 (36.21)** | **0.01** | **0.012** |
| LLB6 | 84.158 (22.26) | 83.748 (22.57) | 0.706 | 0.881 |
| LB1+2+3 | 70.632 (39.46) | 77.401 (33.51) | 0.405 | 0.465 |
| LB1+2 | 70.941 (38.32) | 60.101 (40.45) | 0.179 | 0.256 |
| **LB4+5** | **97.224 (34.52)** | **77.587 (34.39)** | **0.005** | **0.009** |
| **LB9+10** | **48.365 (24.43)** | **63.569 (32.27)** | **0.02** | **0.04** |
| LB1 | 54.318 (26.65) | 53.929 (29.29) | 0.053 | 0.062 |
| LB2 | 86.077 (34.88) | 73.505 (39.31) | 0.061 | 0.098 |
| **LB3** | **72.407 (30.7)** | **65.984 (27.7)** | **0.026** | **0.032** |
| **LB4** | **77.279 (25.53)** | **94.9 (23.96)** | **0.000004** | **0.000008** |
| **LB5** | **80.181 (38.07)** | **108.828 (35.32)** | **0.000003** | **0.000006** |
| LB6 | 77.954 (28.41) | 68.46 (31.39) | 0.087 | 0.173 |
| LB8 | 73.619 (39.11) | 66.844 (29.99) | 0.416 | 0.604 |
| LB9 | 82.096 (35.38) | 76.006 (31.71) | 0.378 | 0.427 |
| LB10 | 69.781 (33.04) | 73.371 (43.51) | 0.634 | 0.847 |
| RLL6 | 69.17 (28.97) | 73.859 (33.16) | 0.385 | 0.77 |
| RLL7 | 71.682 (25.63) | 65.355 (25.14) | 0.182 | 0.339 |
| RB4+5 | 89.483 (37.88) | 78.48 (37.9) | 0.128 | 0.203 |
| **RB9+10** | **70.479 (36.27)** | **93.724 (42.07)** | **0.005** | **0.011** |
| RB1 | 72.393 (37.25) | 60.826 (34.66) | 0.063 | 0.08 |
| **RB2** | **76.531 (37.93)** | **65.423 (24.02)** | **0.019** | **0.028** |
| **RB3** | **75.07 (27.52)** | **89.258 (34.47)** | **0.000495** | **0.000991** |
| **RB4** | **58.086 (23.47)** | **72.085 (23.12)** | **0.000045** | **0.000067** |
| **RB5** | **92.647 (38.92)** | **69.981 (40.61)** | **0.000087** | **0.000173** |
| **RB6** | **62.852 (29.17)** | **82.207 (36.14)** | **0.000414** | **0.000828** |
| RB7 | 85.208 (30.13) | 82.381 (37.11) | 0.686 | 0.701 |
| RB8 | 71.063 (36.99) | 71.216 (31.01) | 0.456 | 0.532 |
| RB9 | 88.757 (44.53) | 92.749 (27.5) | 0.503 | 0.577 |
| **RB10** | **69.437 (35.92)** | **81.238 (39.95)** | **0.019** | **0.037** |
| **Hydraulic diameter, D_h_** | | | | |
| Trachea | 18.306 (2.25) | 18.086 (2.12) | 0.157 | 0.255 |
| LMB | 12.82 (1.77) | 12.871 (1.95) | 0.866 | 0.952 |
| RMB | 14.654 (2.44) | 14.529 (2.23) | 0.603 | 0.702 |
| Bronint | 11.093 (1.47) | 11.356 (1.81) | 0.424 | 0.627 |
| TriLUL | 10.436 (2.44) | 11.18 (2.78) | 0.137 | 0.273 |
| **TriLLB** | **8.539 (1.92)** | **9.466 (2.42)** | **0.018** | **0.035** |
| TriRUL | 12.071 (3.52) | 11.796 (3.75) | 0.606 | 0.745 |
| TriRLL | 7.296 (2.4) | 6.806 (1.71) | 0.259 | 0.291 |
| sLUL | 5.132 (1.97) | 5.158 (1.74) | 0.967 | 1 |
| sLLL | 6.749 (2.14) | 7.025 (1.77) | 0.543 | 0.879 |
| sRUL | 6.587 (2.1) | 6.72 (2.11) | 0.884 | 0.927 |
| sRML | 5.624 (2.15) | 5.824 (3.14) | 0.9 | 0.917 |
| sRLL | 5.249 (1.38) | 5.32 (1.34) | 0.555 | 0.623 |
| LLB6 | 11.595 (3.1) | 11.093 (2.8) | 0.315 | 0.508 |
| LB1+2+3 | 7.138 (2.53) | 8.333 (2.5) | 0.058 | 0.121 |
| **LB1+2** | **4.792 (1.59)** | **5.578 (1.87)** | **0.013** | **0.026** |
| LB4+5 | 8.195 (3.25) | 7.359 (3.4) | 0.23 | 0.46 |
| LB9+10 | 8.983 (2.7) | 7.947 (2.19) | 0.063 | 0.127 |
| LB1 | 4.805 (2.21) | 4.156 (1.27) | 0.176 | 0.235 |
| **LB2** | **4.061 (1.67)** | **4.57 (1.45)** | **0.011** | **0.031** |
| **LB3** | **5.933 (3.61)** | **7.433 (4.98)** | **0.03** | **0.043** |
| LB4 | 5.908 (4.12) | 5.277 (2.27) | 0.378 | 0.448 |
| LB5 | 4.84 (2.62) | 4.605 (2.04) | 0.65 | 0.758 |
| LB6 | 8.921 (4.35) | 9.811 (3.93) | 0.163 | 0.344 |
| LB8 | 6.908 (2.36) | 7.631 (2.76) | 0.262 | 0.364 |
| LB9 | 5.37 (1.79) | 5.323 (1.99) | 0.843 | 0.85 |
| LB10 | 5.49 (2.12) | 5.336 (2.11) | 0.707 | 0.866 |
| RLL6 | 10.424 (2.5) | 10.225 (2.47) | 0.545 | 0.617 |
| RLL7 | 8.142 (1.67) | 8.773 (3.2) | 0.504 | 0.591 |
| RB4+5 | 8.222 (3.28) | 9.613 (3.15) | 0.065 | 0.131 |
| RB9+10 | 5.207 (1.64) | 5.578 (2.1) | 0.413 | 0.531 |
| RB1 | 6.809 (2.92) | 6.888 (3.19) | 0.946 | 1 |
| RB2 | 6.14 (2.71) | 5.921 (3.11) | 0.568 | 0.696 |
| RB3 | 6.819 (3.63) | 7.509 (3.11) | 0.169 | 0.381 |
| RB4 | 6.057 (2.49) | 6.12 (3.41) | 0.885 | 0.964 |
| RB5 | 5.191 (2.33) | 5.527 (3.45) | 0.9 | 0.938 |
| RB6 | 7.395 (2.68) | 7.97 (2.35) | 0.363 | 0.727 |
| RB7 | 6.103 (2.18) | 6.638 (3.42) | 0.65 | 0.816 |
| RB8 | 4.891 (2.42) | 4.087 (1.34) | 0.056 | 0.112 |
| RB9 | 3.899 (1.43) | 3.83 (1.62) | 0.844 | 0.997 |
| RB10 | 3.895 (1.32) | 3.675 (1.37) | 0.418 | 0.697 |
| **Wall thickness, WT** | | | | |
| Trachea | 6.286 (0.55) | 6.266 (0.48) | 0.668 | 0.715 |
| LMB | 6.658 (0.51) | 6.621 (0.49) | 0.169 | 0.295 |
| RMB | 6.903 (0.5) | 6.817 (0.49) | 0.164 | 0.329 |
| Bronint | 5.971 (0.58) | 6.014 (0.65) | 0.692 | 0.822 |
| TriLUL | 6.436 (0.6) | 6.634 (0.57) | 0.08 | 0.16 |
| TriLLB | 5.544 (0.91) | 5.682 (0.7) | 0.809 | 0.959 |
| TriRUL | 6.191 (0.75) | 6.11 (0.66) | 0.391 | 0.445 |
| TriRLL | 4.883 (0.98) | 4.724 (0.72) | 0.208 | 0.416 |
| sLUL | 3.993 (0.78) | 3.956 (0.56) | 0.468 | 0.594 |
| sLLL | 4.668 (0.89) | 4.655 (0.8) | 0.864 | 0.962 |
| sRUL | 4.661 (0.85) | 4.623 (0.75) | 0.572 | 0.783 |
| sRML | 4.138 (0.9) | 3.958 (0.93) | 0.321 | 0.345 |
| sRLL | 4.161 (0.79) | 4.014 (0.65) | 0.162 | 0.323 |
| LLB6 | 6.212 (0.57) | 6.037 (0.79) | 0.223 | 0.247 |
| **LB1+2+3** | **5.447 (0.9)** | **5.868 (0.86)** | **0.028** | **0.036** |
| LB1+2 | 4.026 (0.86) | 4.288 (0.81) | 0.09 | 0.18 |
| LB4+5 | 5.375 (0.99) | 5.166 (0.82) | 0.327 | 0.601 |
| LB9+10 | 5.11 (0.83) | 5.179 (0.92) | 0.71 | 0.964 |
| LB1 | 3.428 (0.83) | 3.445 (0.81) | 0.555 | 0.777 |
| LB2 | 3.473 (0.86) | 3.711 (0.95) | 0.29 | 0.368 |
| LB3 | 4.427 (1.22) | 4.642 (1.2) | 0.445 | 0.77 |
| LB4 | 4.113 (1.19) | 3.92 (0.85) | 0.169 | 0.464 |
| LB5 | 3.948 (0.88) | 4.13 (1.02) | 0.069 | 0.093 |
| LB6 | 5.577 (1.41) | 5.649 (1.14) | 0.857 | 0.983 |
| LB8 | 4.945 (1.06) | 5.218 (1.13) | 0.102 | 0.187 |
| LB9 | 4.056 (1.08) | 4.032 (1.08) | 0.832 | 0.854 |
| LB10 | 3.968 (0.8) | 3.723 (1.03) | 0.182 | 0.363 |
| RLL6 | 5.952 (0.66) | 5.822 (0.7) | 0.13 | 0.181 |
| RLL7 | 5.532 (0.83) | 5.327 (0.87) | 0.107 | 0.169 |
| RB4+5 | 5.449 (1.07) | 5.709 (0.96) | 0.198 | 0.397 |
| RB9+10 | 3.994 (0.97) | 4.107 (0.84) | 0.447 | 0.618 |
| RB1 | 4.531 (1.12) | 4.476 (0.85) | 0.736 | 0.853 |
| RB2 | 4.571 (1.12) | 4.351 (1.15) | 0.232 | 0.458 |
| RB3 | 4.796 (0.92) | 5.167 (0.95) | 0.075 | 0.101 |
| RB4 | 4.213 (1.2) | 4.099 (0.93) | 0.613 | 0.761 |
| RB5 | 4.062 (0.95) | 3.817 (1.2) | 0.09 | 0.191 |
| RB6 | 4.802 (1.12) | 5.144 (0.71) | 0.347 | 0.382 |
| RB7 | 4.663 (1.12) | 4.471 (1.11) | 0.305 | 0.609 |
| **RB8** | **4.1 (1.22)** | **3.451 (0.93)** | **0.001** | **0.002** |
| **RB9** | **3.654 (0.81)** | **3.297 (0.87)** | **0.021** | **0.043** |
| RB10 | 3.364 (0.93) | 3.392 (0.94) | 0.885 | 0.945 |
| **Circularity, Cr** | | | | |
| Trachea | 0.994 (0.01) | 0.995 (0) | 0.357 | 0.418 |
| LMB | 0.984 (0.02) | 0.982 (0.01) | 0.421 | 0.48 |
| RMB | 0.967 (0.01) | 0.968 (0.01) | 0.778 | 0.95 |
| Bronint | 0.969 (0.04) | 0.982 (0.02) | 0.116 | 0.231 |
| TriLUL | 0.955 (0.03) | 0.951 (0.04) | 0.794 | 0.868 |
| TriLLB | 0.976 (0.02) | 0.969 (0.03) | 0.069 | 0.084 |
| TriRUL | 0.91 (0.04) | 0.904 (0.06) | 0.582 | 0.664 |
| TriRLL | 0.965 (0.02) | 0.969 (0.03) | 0.224 | 0.518 |
| sLUL | 0.921 (0.03) | 0.927 (0.03) | 0.224 | 0.366 |
| sLLL | 0.944 (0.02) | 0.938 (0.02) | 0.272 | 0.545 |
| sRUL | 0.929 (0.03) | 0.917 (0.03) | 0.199 | 0.329 |
| sRML | 0.915 (0.04) | 0.922 (0.06) | 0.458 | 0.673 |
| sRLL | 0.936 (0.02) | 0.939 (0.03) | 0.633 | 0.897 |
| LLB6 | 0.953 (0.03) | 0.946 (0.03) | 0.357 | 0.602 |
| LB1+2+3 | 0.958 (0.05) | 0.961 (0.04) | 0.771 | 0.897 |
| LB1+2 | 0.959 (0.03) | 0.957 (0.05) | 0.779 | 0.92 |
| LB4+5 | 0.919 (0.07) | 0.918 (0.06) | 0.96 | 0.974 |
| LB9+10 | 0.957 (0.03) | 0.962 (0.02) | 0.466 | 0.53 |
| LB1 | 0.924 (0.06) | 0.94 (0.04) | 0.161 | 0.286 |
| LB2 | 0.908 (0.05) | 0.919 (0.07) | 0.455 | 0.628 |
| LB3 | 0.928 (0.05) | 0.918 (0.06) | 0.182 | 0.202 |
| LB4 | 0.902 (0.05) | 0.911 (0.06) | 0.09 | 0.095 |
| LB5 | 0.936 (0.08) | 0.934 (0.05) | 0.111 | 0.233 |
| **LB6** | **0.921 (0.05)** | **0.891 (0.05)** | **0.017** | **0.034** |
| LB8 | 0.949 (0.03) | 0.952 (0.03) | 0.74 | 0.769 |
| LB9 | 0.947 (0.05) | 0.948 (0.03) | 0.987 | 1 |
| LB10 | 0.958 (0.03) | 0.961 (0.03) | 0.915 | 0.949 |
| RLL6 | 0.968 (0.02) | 0.96 (0.03) | 0.41 | 0.475 |
| RLL7 | 0.966 (0.04) | 0.968 (0.02) | 0.456 | 0.665 |
| RB4+5 | 0.939 (0.04) | 0.924 (0.06) | 0.231 | 0.461 |
| RB9+10 | 0.954 (0.04) | 0.952 (0.06) | 0.692 | 0.937 |
| RB1 | 0.92 (0.07) | 0.919 (0.08) | 0.779 | 0.866 |
| **RB2** | **0.943 (0.03)** | **0.923 (0.04)** | **0.029** | **0.059** |
| RB3 | 0.924 (0.06) | 0.908 (0.05) | 0.066 | 0.072 |
| **RB4** | **0.909 (0.07)** | **0.926 (0.06)** | **0.048** | **0.052** |
| RB5 | 0.921 (0.05) | 0.918 (0.08) | 0.992 | 1 |
| RB6 | 0.93 (0.05) | 0.926 (0.04) | 0.683 | 0.761 |
| RB7 | 0.942 (0.04) | 0.94 (0.05) | 0.824 | 0.959 |
| **RB8** | **0.918 (0.06)** | **0.955 (0.03)** | **0.0007** | **0.0014** |
| RB9 | 0.94 (0.04) | 0.931 (0.06) | 0.327 | 0.408 |
| RB10 | 0.953 (0.05) | 0.938 (0.05) | 0.066 | 0.149 |

Supplementary Table 4. Comparison of blood vessel volumes and total blood vessel volumes of each region extracted from images. WL, whole lung; RL, right lung; LL, left lung; LUL, left upper lobe; LLL, left lower lobe; RUL, right upper lobe; RML, right middle lobe; RLL, right lower lobe; BV, blood vessel volume; TBV, total blood volume.

| **Region** | **Baseline** | **One-year later** | **p-value** | **Q-value** |
| --- | --- | --- | --- | --- |
| **BV1** | | | |  |
| LLL | 0.404 (0.36) | 0.361 (0.3) | 0.938 | 0.946 |
| LUL | 0.367 (0.33) | 0.322 (0.21) | 0.308 | 0.318 |
| RLL | 0.596 (0.57) | 0.522 (0.39) | 0.544 | 0.555 |
| RML | 0.113 (0.15) | 0.093 (0.14) | 0.318 | 0.4 |
| RUL | 0.308 (0.33) | 0.249 (0.24) | 0.468 | 0.48 |
| LL | 0.771 (0.6) | 0.683 (0.41) | 0.433 | 0.444 |
| RL | 1.017 (0.87) | 0.864 (0.67) | 0.232 | 0.239 |
| WL | 1.788 (1.43) | 1.547 (1.02) | 0.299 | 0.308 |
| **BV2** | | | | |
| LLL | 2.025 (1.98) | 1.756 (1.43) | 0.583 | 0.595 |
| LUL | 1.726 (1.49) | 1.546 (1.01) | 0.638 | 0.65 |
| RLL | 2.833 (2.8) | 2.527 (1.91) | 0.953 | 0.961 |
| RML | 0.555 (0.75) | 0.47 (0.67) | 0.481 | 0.492 |
| RUL | 1.484 (1.52) | 1.187 (1.12) | 0.445 | 0.456 |
| LL | 3.75 (3.03) | 3.302 (2) | 0.652 | 0.664 |
| RL | 4.872 (4.18) | 4.183 (3.25) | 0.583 | 0.595 |
| WL | 8.622 (7.09) | 7.485 (5.05) | 0.597 | 0.608 |
| **BV3** | | | | |
| LLL | 3.853 (3.46) | 3.306 (2.54) | 0.505 | 0.517 |
| LUL | 3.375 (2.49) | 2.884 (1.65) | 0.256 | 0.264 |
| RLL | 5.397 (4.7) | 4.639 (3.22) | 0.57 | 0.581 |
| RML | 1.1 (1.45) | 0.86 (1.09) | 0.203 | 0.209 |
| RUL | 2.769 (2.52) | 2.213 (1.84) | 0.337 | 0.347 |
| LL | 7.228 (5.14) | 6.19 (3.36) | 0.299 | 0.308 |
| RL | 9.265 (7.08) | 7.712 (5.24) | 0.308 | 0.318 |
| WL | 16.494 (11.98) | 13.902 (8.23) | 0.232 | 0.239 |
| **BV4** | | | | |
| LLL | 6.153 (5.42) | 5.43 (4.2) | 0.695 | 0.706 |
| LUL | 5.425 (3.86) | 4.764 (2.85) | 0.224 | 0.232 |
| RLL | 8.581 (7.14) | 7.491 (5.16) | 0.597 | 0.608 |
| RML | 1.798 (2.3) | 1.472 (1.87) | 0.281 | 0.29 |
| RUL | 4.466 (4.04) | 3.667 (3.18) | 0.41 | 0.421 |
| LL | 11.578 (7.92) | 10.194 (5.71) | 0.367 | 0.378 |
| RL | 14.845 (10.93) | 12.63 (8.76) | 0.357 | 0.367 |
| WL | 26.423 (18.46) | 22.824 (13.88) | 0.357 | 0.367 |
| **BV5** | | | | |
| LLL | 8.262 (6.86) | 7.289 (5.47) | 0.61 | 0.622 |
| LUL | 7.46 (5.03) | 6.517 (3.76) | 0.281 | 0.29 |
| RLL | 11.578 (9.04) | 10.064 (6.69) | 0.638 | 0.65 |
| RML | 2.461 (3.06) | 1.983 (2.41) | 0.29 | 0.299 |
| RUL | 5.968 (5.18) | 4.942 (4.21) | 0.481 | 0.492 |
| LL | 15.722 (10.08) | 13.806 (7.4) | 0.41 | 0.421 |
| RL | 20.007 (13.95) | 16.989 (11.37) | 0.29 | 0.299 |
| WL | 35.729 (23.5) | 30.795 (17.98) | 0.327 | 0.337 |
| **BV6** | | | | |
| LLL | 10.465 (8.47) | 9.214 (6.87) | 0.518 | 0.529 |
| LUL | 9.494 (6.23) | 8.239 (4.73) | 0.217 | 0.224 |
| RLL | 14.556 (10.88) | 12.717 (8.25) | 0.433 | 0.444 |
| RML | 3.128 (3.76) | 2.53 (2.85) | 0.24 | 0.247 |
| RUL | 7.501 (6.37) | 6.204 (5.2) | 0.308 | 0.318 |
| LL | 19.959 (12.35) | 17.453 (9.31) | 0.248 | 0.256 |
| RL | 25.185 (16.84) | 21.451 (13.83) | 0.256 | 0.264 |
| WL | 45.144 (28.53) | 38.904 (22.19) | 0.189 | 0.195 |
| **BV7** | | | | |
| LLL | 12.798 (10.1) | 11.334 (8.31) | 0.681 | 0.692 |
| LUL | 11.61 (7.48) | 10.176 (5.87) | 0.164 | 0.169 |
| RLL | 17.667 (12.73) | 15.684 (9.95) | 0.681 | 0.692 |
| RML | 3.863 (4.5) | 3.173 (3.46) | 0.248 | 0.256 |
| RUL | 9.155 (7.73) | 7.655 (6.47) | 0.399 | 0.41 |
| LL | 24.409 (14.54) | 21.511 (11.31) | 0.264 | 0.272 |
| RL | 30.686 (19.87) | 26.512 (16.85) | 0.378 | 0.388 |
| WL | 55.094 (33.62) | 48.022 (27.02) | 0.337 | 0.347 |
| **BV8** | | | | |
| LLL | 15.065 (11.49) | 13.328 (9.7) | 0.433 | 0.444 |
| LUL | 13.584 (8.53) | 12.023 (6.74) | 0.147 | 0.152 |
| RLL | 20.668 (14.29) | 18.375 (11.38) | 0.445 | 0.456 |
| RML | 4.572 (5.06) | 3.784 (3.92) | 0.21 | 0.217 |
| RUL | 10.662 (8.7) | 8.99 (7.6) | 0.29 | 0.299 |
| LL | 28.649 (16.53) | 25.351 (12.97) | 0.272 | 0.281 |
| RL | 35.901 (22.14) | 31.149 (19.28) | 0.232 | 0.239 |
| WL | 64.55 (37.75) | 56.5 (30.94) | 0.183 | 0.189 |
| **BV9** | | | | |
| LLL | 17.163 (12.73) | 15.028 (10.75) | 0.389 | 0.399 |
| LUL | 15.486 (9.54) | 13.625 (7.49) | 0.085 | 0.087 |
| RLL | 23.398 (15.62) | 20.698 (12.36) | 0.378 | 0.388 |
| RML | 5.251 (5.62) | 4.325 (4.29) | 0.183 | 0.189 |
| RUL | 12.072 (9.65) | 10.222 (8.56) | 0.281 | 0.29 |
| LL | 32.649 (18.27) | 28.653 (14.2) | 0.164 | 0.169 |
| RL | 40.72 (24.28) | 35.245 (21.03) | 0.217 | 0.224 |
| WL | 73.369 (41.51) | 63.898 (33.75) | 0.131 | 0.135 |
| **BV10** | | | | |
| LLL | 19.079 (13.92) | 16.936 (11.94) | 0.468 | 0.48 |
| LUL | 17.363 (10.32) | 15.317 (8.21) | 0.06 | 0.061 |
| RLL | 25.931 (16.83) | 23.357 (13.74) | 0.695 | 0.706 |
| RML | 5.821 (6.03) | 5.006 (4.87) | 0.367 | 0.378 |
| RUL | 13.457 (10.61) | 11.529 (9.6) | 0.357 | 0.367 |
| LL | 36.442 (19.66) | 32.253 (15.67) | 0.189 | 0.195 |
| RL | 45.209 (26.08) | 39.892 (23.41) | 0.299 | 0.308 |
| WL | 81.651 (44.56) | 72.145 (37.43) | 0.147 | 0.152 |
| **BV11** | | | | |
| LLL | 21.25 (15.28) | 18.786 (13.28) | 0.399 | 0.41 |
| **LUL** | **19.207 (11.46)** | **16.974 (9.12)** | **0.036** | **0.045** |
| RLL | 28.845 (18.25) | 25.919 (14.99) | 0.41 | 0.421 |
| RML | 6.569 (6.7) | 5.596 (5.27) | 0.256 | 0.264 |
| RUL | 14.85 (11.69) | 12.652 (10.49) | 0.281 | 0.29 |
| LL | 40.457 (21.37) | 35.759 (17.33) | 0.126 | 0.13 |
| RL | 50.263 (28.42) | 44.166 (25.32) | 0.117 | 0.12 |
| WL | 90.72 (48.52) | 79.926 (40.8) | 0.147 | 0.152 |
| **BV12** | | | | |
| LLL | 23.109 (16.33) | 20.261 (14.15) | 0.41 | 0.421 |
| **LUL** | **20.851 (12.17)** | **18.547 (9.66)** | **0.048** | **0.05** |
| RLL | 31.305 (19.15) | 28.17 (15.77) | 0.256 | 0.264 |
| RML | 7.075 (7.09) | 6.113 (5.6) | 0.281 | 0.29 |
| RUL | 15.993 (12.36) | 13.712 (11.23) | 0.318 | 0.327 |
| LL | 43.96 (22.55) | 38.808 (18.03) | 0.1 | 0.102 |
| RL | 54.373 (29.65) | 47.996 (26.57) | 0.108 | 0.111 |
| WL | 98.333 (50.77) | 86.803 (42.59) | 0.1 | 0.102 |
| **BV13** | | | | |
| LLL | 24.726 (17.36) | 21.578 (15.04) | 0.347 | 0.357 |
| **LUL** | **22.306 (12.82)** | **19.838 (10.29)** | **0.04** | **0.048** |
| RLL | 33.603 (20.09) | 30.179 (16.56) | 0.217 | 0.224 |
| RML | 7.559 (7.48) | 6.538 (5.93) | 0.299 | 0.308 |
| RUL | 17.02 (13.08) | 14.623 (11.93) | 0.308 | 0.318 |
| LL | 47.033 (23.78) | 41.416 (18.99) | 0.063 | 0.063 |
| RL | 58.182 (31.07) | 51.34 (27.8) | 0.096 | 0.098 |
| WL | 105.214 (53.28) | 92.757 (44.63) | 0.075 | 0.076 |
| **BV14** | | | | |
| LLL | 26.095 (18.23) | 22.874 (15.89) | 0.422 | 0.433 |
| **LUL** | **23.614 (13.4)** | **21.112 (10.87)** | **0.046** | **0.049** |
| RLL | 35.573 (20.72) | 32.138 (17.2) | 0.272 | 0.281 |
| RML | 7.984 (7.83) | 6.953 (6.25) | 0.399 | 0.41 |
| RUL | 18.01 (13.8) | 15.555 (12.66) | 0.389 | 0.399 |
| LL | 49.708 (24.75) | 43.986 (19.91) | 0.068 | 0.069 |
| RL | 61.567 (32.09) | 54.645 (28.98) | 0.117 | 0.12 |
| WL | 111.276 (55.13) | 98.631 (46.51) | 0.1 | 0.102 |
| **BV15** | | | | |
| LLL | 27.296 (19.02) | 23.934 (16.66) | 0.422 | 0.433 |
| **LUL** | **24.674 (13.9)** | **22.109 (11.33)** | **0.046** | **0.049** |
| RLL | 37.263 (21.29) | 33.682 (17.73) | 0.217 | 0.224 |
| RML | 8.324 (8.1) | 7.26 (6.46) | 0.399 | 0.41 |
| RUL | 18.739 (14.31) | 16.241 (13.19) | 0.399 | 0.41 |
| LL | 51.97 (25.69) | 46.043 (20.68) | 0.071 | 0.073 |
| RL | 64.327 (32.9) | 57.183 (29.85) | 0.1 | 0.102 |
| WL | 116.297 (56.75) | 103.226 (47.96) | 0.092 | 0.094 |
| **BV16** | | | | |
| LLL | 28.285 (19.61) | 24.765 (17.22) | 0.445 | 0.456 |
| **LUL** | **25.583 (14.27)** | **22.94 (11.72)** | **0.042** | **0.048** |
| RLL | 38.682 (21.75) | 34.992 (18.11) | 0.21 | 0.217 |
| RML | 8.613 (8.3) | 7.498 (6.6) | 0.399 | 0.41 |
| RUL | 19.343 (14.72) | 16.806 (13.63) | 0.41 | 0.421 |
| LL | 53.869 (26.34) | 47.705 (21.24) | 0.052 | 0.053 |
| RL | 66.637 (33.52) | 59.296 (30.49) | 0.081 | 0.083 |
| WL | 120.506 (57.93) | 107.001 (49.01) | 0.096 | 0.098 |
| **BV17** | | | | |
| LLL | 29.06 (20.06) | 25.437 (17.69) | 0.399 | 0.41 |
| **LUL** | **26.28 (14.59)** | **23.584 (12.01)** | **0.041** | **0.048** |
| RLL | 39.784 (21.97) | 36.036 (18.36) | 0.217 | 0.224 |
| RML | 8.797 (8.44) | 7.671 (6.7) | 0.41 | 0.421 |
| RUL | 19.796 (15.05) | 17.231 (13.93) | 0.445 | 0.456 |
| LL | 55.34 (26.88) | 49.022 (21.67) | 0.052 | 0.053 |
| RL | 68.378 (33.9) | 60.938 (30.88) | 0.1 | 0.102 |
| WL | 123.718 (58.73) | 109.96 (49.72) | 0.085 | 0.087 |
| **BV18** | | | | |
| LLL | 29.759 (20.5) | 25.962 (18.08) | 0.347 | 0.357 |
| **LUL** | **26.862 (14.85)** | **24.116 (12.27)** | **0.038** | **0.048** |
| RLL | 40.776 (22.24) | 36.927 (18.64) | 0.203 | 0.209 |
| RML | 8.965 (8.56) | 7.81 (6.78) | 0.389 | 0.399 |
| RUL | 20.24 (15.34) | 17.608 (14.22) | 0.41 | 0.421 |
| **LL** | **56.62 (27.4)** | **50.078 (22.12)** | **0.046** | **0.046** |
| RL | 69.981 (34.27) | 62.344 (31.33) | 0.085 | 0.087 |
| WL | 126.601 (59.51) | 112.422 (50.54) | 0.065 | 0.066 |
| **BV19** | | | | |
| LLL | 30.295 (20.84) | 26.379 (18.36) | 0.308 | 0.318 |
| **LUL** | **27.309 (15.07)** | **24.535 (12.43)** | **0.039** | **0.048** |
| RLL | 41.503 (22.45) | 37.622 (18.79) | 0.189 | 0.195 |
| RML | 9.083 (8.66) | 7.913 (6.85) | 0.389 | 0.399 |
| RUL | 20.563 (15.58) | 17.88 (14.4) | 0.399 | 0.41 |
| **LL** | **57.604 (27.81)** | **50.914 (22.35)** | **0.04** | **0.048** |
| RL | 71.149 (34.59) | 63.416 (31.54) | 0.075 | 0.076 |
| WL | 128.753 (60.18) | 114.33 (50.89) | 0.055 | 0.055 |
| **BV20** | | | | |
| LLL | 30.678 (21.07) | 26.705 (18.56) | 0.29 | 0.299 |
| **LUL** | **27.673 (15.23)** | **24.877 (12.56)** | **0.038** | **0.048** |
| RLL | 42.126 (22.58) | 38.193 (18.94) | 0.203 | 0.209 |
| RML | 9.188 (8.73) | 8.017 (6.92) | 0.399 | 0.41 |
| RUL | 20.879 (15.8) | 18.106 (14.56) | 0.399 | 0.41 |
| **LL** | **58.352 (28.07)** | **51.582 (22.54)** | **0.036** | **0.038** |
| RL | 72.192 (34.81) | 64.316 (31.8) | 0.081 | 0.083 |
| WL | 130.544 (60.58) | 115.898 (51.29) | 0.055 | 0.055 |
| **TBV** | | | | |
| LLL | 32.526 (22.15) | 28.186 (19.64) | 0.256 | 0.264 |
| **LUL** | **28.989 (15.77)** | **26.185 (13.18)** | **0.045** | **0.049** |
| RLL | 45.236 (23.48) | 41.32 (19.86) | 0.177 | 0.224 |
| RML | 9.723 (9.09) | 8.492 (7.25) | 0.389 | 0.399 |
| RUL | 21.858 (16.37) | 18.898 (15.06) | 0.389 | 0.399 |
| **LL** | **61.514 (28.97)** | **54.371 (23.43)** | **0.031** | **0.035** |
| RL | 76.817 (35.57) | 68.71 (32.79) | 0.055 | 0.055 |
| **WL** | **138.332 (61.95)** | **123.08 (52.68)** | **0.031** | **0.043** |

Supplementary Table 5. The QCT-based airway structural variables of subjects extracted from original (LR) and generated (SR) images at baseline and one-year follow-up. Angle, θ; hydraulic diameter, D_h_; wall thickness, WT; circularity, Cr; left main bronchus, LMB; right main bronchus, RMB; trifurcation of the left lower lobe, TriLLB; trifurcation of the left upper lobe, TriLUL; trifurcation of the right lower lobe, TriRLL; trifurcation of the right upper lobe, TriRUL; subgroup right upper lobe, sRUL; subgroup right middle lobe, sRML; subgroup right lower lobe, sRLL; subgroup left upper lobe, sLUL; subgroup left lower lobe, sLLL. Values are presented as means (standard deviation).

| **Region** | **LR** | | | **SR** | | |
| --- | --- | --- | --- | --- | --- | --- |
|  | **Baseline** | **One-year later** | **p-value** | **Baseline** | **One-year later** | **p-value** |
| **θ** | | | | | | |
| Trachea | 84.656 (13.3) | 83.714 (11.79) | 0.50 | 84.462 (13.46) | 83.321 (11.64) | 0.46 |
| LMB | 85.586 (15.42) | 86.967 (17.99) | 0.66 | 86.358 (15.4) | 88.635 (20.44) | 0.38 |
| RMB | 101.455 (16.62) | 104.408 (17.32) | 0.60 | 101.386 (17.124) | 104.149 (17.68) | 0.62 |
| Bronint | 66.857 (26.14) | 66.569 (22.96) | 0.85 | 64.949 (25.847) | 62.284 (20.37) | 0.79 |
| TriLUL | 104.293 (23.07) | 112.183 (24.38) | 0.59 | 106.733 (26.572) | 107.403 (24.35) | 0.49 |
| TriLLB | 58.74 (18.43) | 56.873 (19.61) | 0.99 | 58.246 (20.002) | 56.963 (20.4) | 0.74 |
| TriRUL | 64.43 (23.05) | 80.13 (29.13) | 0.30 | 72.159 (24.497) | 80.055 (28.15) | 0.14 |
| **TriRLL** | **65.206 (27.46)** | **50.927 (26.4)** | **0.02** | **67.555 (27.469)** | **54.54 (36.21)** | **0.01** |
| **D_h_** | | | | | | |
| Trachea | 18.277 (2.29) | 18.023 (2.13) | 0.14 | 18.306 (2.249) | 18.086 (2.12) | 0.16 |
| LMB | 12.767 (1.75) | 12.698 (1.81) | 0.81 | 12.82 (1.771) | 12.871 (1.95) | 0.87 |
| RMB | 14.531 (2.43) | 14.608 (2.2) | 0.70 | 14.654 (2.442) | 14.529 (2.23) | 0.60 |
| Bronint | 11.081 (1.49) | 11.197 (1.52) | 0.46 | 11.093 (1.466) | 11.356 (1.81) | 0.42 |
| TriLUL | 10.648 (2.64) | 10.995 (2.62) | 0.72 | 10.436 (2.439) | 11.18 (2.78) | 0.14 |
| **TriLLB** | 8.498 (1.97) | 9.161 (2.19) | 0.07 | **8.539 (1.924)** | **9.466 (2.42)** | **0.02** |
| TriRUL | 12.046 (3.74) | 11.573 (3.26) | 0.38 | 12.071 (3.519) | 11.796 (3.75) | 0.61 |
| TriRLL | 7.263 (2.36) | 6.596 (1.91) | 0.20 | 7.296 (2.403) | 6.806 (1.71) | 0.26 |
| sLUL | 5.204 (1.92) | 5.139 (1.48) | 0.63 | 5.132 (1.974) | 5.158 (1.74) | 0.97 |
| sLLL | 6.804 (2.02) | 6.938 (1.62) | 0.98 | 6.749 (2.143) | 7.025 (1.77) | 0.54 |
| sRUL | 7.177 (2.28) | 6.937 (2.22) | 0.82 | 6.587 (2.098) | 6.72 (2.11) | 0.88 |
| sRML | 5.316 (1.94) | 5.295 (1.98) | 0.37 | 5.624 (2.151) | 5.824 (3.14) | 0.9 |
| sRLL | 5.324 (1.44) | 5.210 (1.28) | 0.50 | 5.249 (1.381) | 5.32 (1.34) | 0.56 |
| **WT** | | | | | | |
| Trachea | 6.331 (0.56) | 6.238 (0.62) | 0.49 | 6.286 (0.546) | 6.266 (0.48) | 0.67 |
| LMB | 6.7 (0.46) | 6.635 (0.67) | 0.21 | 6.658 (0.507) | 6.621 (0.49) | 0.17 |
| RMB | 6.939 (0.45) | 6.742 (0.74) | 0.08 | 6.903 (0.497) | 6.817 (0.49) | 0.16 |
| Bronint | 6.025 (0.57) | 5.891 (0.71) | 0.38 | 5.971 (0.581) | 6.014 (0.65) | 0.69 |
| TriLUL | 6.449 (0.65) | 6.528 (0.68) | 0.69 | 6.436 (0.604) | 6.634 (0.57) | 0.08 |
| TriLLB | 5.646 (0.94) | 5.63 (0.78) | 0.90 | 5.544 (0.909) | 5.682 (0.7) | 0.81 |
| TriRUL | 6.187 (0.82) | 6.006 (0.8) | 0.12 | 6.191 (0.749) | 6.11 (0.66) | 0.39 |
| TriRLL | 5.004 (0.89) | 4.821 (0.93) | 0.18 | 4.883 (0.977) | 4.724 (0.72) | 0.21 |
| sLUL | 4.04 (0.76) | 3.793 (0.59) | 0.07 | 3.993 (0.784) | 3.956 (0.56) | 0.47 |
| sLLL | 4.726 (0.81) | 4.626 (0.77) | 0.36 | 4.668 (0.886) | 4.655 (0.8) | 0.86 |
| sRUL | 4.757 (0.84) | 4.628 (0.8) | 0.27 | 4.661 (0.854) | 4.623 (0.75) | 0.57 |
| sRML | 4.019 (0.88) | 3.834 (0.87) | 0.45 | 4.138 (0.898) | 3.958 (0.93) | 0.32 |
| sRLL | 4.208 (0.75) | 4.045 (0.71) | 0.09 | 4.161 (0.794) | 4.014 (0.65) | 0.16 |
| **Cr** | | | | | | |
| Trachea | 0.994 (0.01) | 0.995 (0.01) | 0.41 | 0.994 (0.009) | 0.995 (0) | 0.36 |
| LMB | 0.984 (0.02) | 0.984 (0.01) | 0.81 | 0.984 (0.015) | 0.982 (0.01) | 0.42 |
| RMB | 0.967 (0.01) | 0.968 (0.01) | 0.51 | 0.967 (0.011) | 0.968 (0.01) | 0.78 |
| Bronint | 0.97 (0.04) | 0.982 (0.02) | 0.12 | 0.969 (0.044) | 0.982 (0.02) | 0.12 |
| TriLUL | 0.95 (0.04) | 0.949 (0.04) | 0.75 | 0.955 (0.033) | 0.951 (0.04) | 0.79 |
| TriLLB | 0.978 (0.02) | 0.971 (0.03) | 0.07 | 0.976 (0.022) | 0.969 (0.03) | 0.07 |
| TriRUL | 0.908 (0.04) | 0.904 (0.05) | 0.67 | 0.91 (0.043) | 0.904 (0.06) | 0.58 |
| TriRLL | 0.968 (0.02) | 0.977 (0.02) | 0.07 | 0.965 (0.022) | 0.969 (0.03) | 0.22 |
| sLUL | 0.95 (0.04) | 0.939 (0.03) | 0.29 | 0.921 (0.03) | 0.927 (0.03) | 0.22 |
| sLLL | 0.94 (0.03) | 0.938 (0.02) | 0.66 | 0.944 (0.022) | 0.938 (0.02) | 0.27 |
| sRUL | 0.922 (0.03) | 0.918 (0.03) | 0.69 | 0.929 (0.031) | 0.917 (0.03) | 0.2 |
| sRML | 0.914 (0.04) | 0.919 (0.06) | 0.79 | 0.915 (0.044) | 0.922 (0.06) | 0.46 |
| sRLL | 0.942 (0.02) | 0.945 (0.02) | 0.41 | 0.936 (0.024) | 0.939 (0.03) | 0.63 |

Supplementary Table 6. QCT-based parenchymal functional variables of subjects extracted from original (LR) and generated (SR) images at baseline and one-year later. Left upper lobe, LUL; left lower lobe, LLL; right upper lobe, RUL; right middle lobe, RML; right lower lobe, RLL; consolidation, Conso; semi consolidation, Semiconso; normal, Norm; emphysema, Emph; ground-glass opacity, GGO; fibrosis, Fibr.

| **Region** | **LR** | | | **SR** | | |
| --- | --- | --- | --- | --- | --- | --- |
|  | **Baseline** | **One-year later** | **p-value** | **Baseline** | **One-year later** | **p-value** |
| **Conso, %** | | | | | | |
| LUL | 2.034 (1.24) | 1.993 (1.15) | 0.98 | 2.779 (1.64) | 2.988 (1.71) | 0.36 |
| LLL | 2.096 (1.00) | 1.993 (0.66) | 0.38 | 2.365 (1.10) | 2.281 (1.01) | 0.57 |
| RUL | 2.503 (1.39) | 2.487 (1.31) | 0.98 | 2.859 (1.33) | 3.237 (1.53) | 0.06 |
| RML | 1.759 (1.27) | 1.838 (1.20) | 0.95 | 1.995 (1.53) | 2.009 (1.39) | 0.57 |
| RLL | 2.653 (1.45) | 2.592 (1.21) | 0.41 | 2.595 (1.41) | 2.592 (1.14) | 0.33 |
| Total | 2.141 (0.93) | 2.056 (0.64) | 0.86 | 2.513 (1.06) | 2.605 (1.05) | 0.20 |
| **Semiconso, %** | | | | | | |
| LUL | 6.102 (3.25) | 5.871 (2.86) | 0.92 | 5.479 (3.53) | 5.385 (3.23) | 0.75 |
| LLL | 8.795 (7.96) | 7.613 (3.66) | 0.42 | 8.191 (8.08) | 6.542 (3.45) | 0.64 |
| RUL | 6.776 (3.01) | 6.456 (2.61) | 0.64 | 5.731 (3.35) | 5.482 (3.16) | 0.72 |
| RML | 5.910 (3.69) | 5.88 (2.92) | 0.31 | 4.990 (3.51) | 4.141 (2.36) | 0.84 |
| RLL | 9.292 (7.89) | 8.132 (4.22) | 0.31 | 8.370 (7.86) | 6.853 (4.01) | 0.54 |
| Total | 7.278 (4.76) | 6.497 (2.27) | 0.86 | 6.539 (4.94) | 5.578 (2.60) | 0.9 |
| **Norm, %** | | | | | | |
| LUL | 71.489 (13.49) | 72.659 (12.31) | 0.56 | 58.012 (25.17) | 55.457 (27.51) | 0.43 |
| LLL | 67.358 (17.73) | 68.634 (11.42) | 0.71 | 59.773 (21.69) | 61.446 (18.81) | 0.82 |
| RUL | 71.076 (11.65) | 71.806 (10.71) | 0.62 | **58.775 (26.25)** | **55.001 (28.85)** | **0.04** |
| RML | 72.509 (12.39) | 73.336 (11.20) | 0.55 | 62.550 (25.23) | 59.713 (26.91) | 0.75 |
| RLL | 67.734 (18.42) | 68.256 (12.68) | 0.24 | 61.552 (21.55) | 61.550 (18.09) | 0.35 |
| Total | 70.762 (12.47) | 71.916 (7.780) | 0.84 | 60.576 (21.72) | 59.078 (22.35) | 0.14 |
| **Emph, %** | | | | | | |
| LUL | 8.396 (11.84) | 7.878 (11.7) | 0.37 | 7.707 (11.3) | 8.035 (11.98) | 0.39 |
| LLL | 4.791 (6.49) | 4.467 (6.30) | 0.47 | 4.859 (6.47) | 4.792 (7.19) | 0.29 |
| RUL | 6.781 (10.05) | 6.851 (10.58) | 0.72 | 5.89 (8.45) | 5.694 (8.76) | 0.29 |
| RML | 9.329 (11.55) | 8.186 (11.23) | 0.58 | 9.344 (10.76) | 10.069 (14.49) | 0.79 |
| RLL | 3.468 (5.75) | 3.551 (5.84) | 0.54 | 4.057 (6.64) | 4.404 (7.67) | 0.71 |
| Total | 5.791 (7.16) | 5.537 (7.34) | 0.21 | 6.016 (7.72) | 6.025 (8.32) | 0.24 |
| **GGO, %** | | | | | | |
| LUL | 11.848 (6.68) | 11.431 (5.23) | 0.70 | 10.269 (7.56) | 9.37 (6.69) | 0.96 |
| LLL | 16.843 (10.88) | 17.122 (8.84) | 0.31 | 14.813 (10.67) | 13.14 (7.97) | 0.96 |
| RUL | 12.749 (6.14) | 12.228 (4.72) | 0.65 | 10.516 (7.36) | 9.565 (6.29) | 0.66 |
| RML | 10.408 (5.32) | 10.647 (4.51) | 0.37 | 8.374 (6.12) | 7.174 (4.83) | 0.92 |
| RLL | 16.723 (10.12) | 17.282 (8.41) | 0.36 | 14.560 (10.17) | 13.959 (9.17) | 0.84 |
| Total | 13.909 (7.57) | 13.826 (5.54) | 0.49 | 12.011 (8.14) | 10.909 (6.67) | 0.92 |
| **Fibr, %** | | | | | | |
| LUL | 7.546 (4.47) | 7.486 (3.82) | 0.78 | 7.664 (4.27) | 7.925 (3.85) | 0.21 |
| LLL | 10.503 (8.72) | 9.189 (4.14) | 0.49 | 9.512 (8.96) | 7.709 (4.01) | 0.47 |
| RUL | 8.677 (4.19) | 8.443 (3.58) | 0.67 | 8.187 (3.58) | 8.303 (3.70) | 0.23 |
| RML | 7.261 (4.70) | 7.405 (3.78) | 0.36 | 7.654 (4.95) | 8.236 (5.52) | 0.35 |
| RLL | 11.417 (9.01) | 10.189 (5.21) | 0.30 | 10.435 (8.97) | 9.031 (4.88) | 0.25 |
| Total | 9.018 (5.45) | 8.133 (2.77) | 1.00 | 8.552 (5.49) | 7.767 (2.81) | 0.64 |

Supplementary Table 7. The whole lung blood vessel volume of subjects extracted from original (LR) and generated (SR) images at baseline and one-year follow-up. Blood vessel volume, BV; total blood volume, TBV.

| **Variable** | **LR** | | | **SR** | | |
| --- | --- | --- | --- | --- | --- | --- |
|  | **Baseline** | **One-year later** | **p-value** | **Baseline** | **One-year later** | **p-value** |
| BV1 | 2.084 (1.66) | 1.915 (1.33) | 0.60 | 1.788 (1.43) | 1.547 (1.02) | 0.30 |
| BV2 | 9.951 (7.98) | 9.235 (6.69) | 0.86 | 8.622 (7.09) | 7.485 (5.05) | 0.60 |
| BV3 | 18.92 (13.55) | 16.935 (10.24) | 0.44 | 16.494 (11.98) | 13.902 (8.23) | 0.23 |
| BV4 | 30.256 (21.11) | 27.603 (17.52) | 0.51 | 26.423 (18.46) | 22.824 (13.88) | 0.36 |
| BV5 | 40.728 (26.80) | 37.082 (22.63) | 0.64 | 35.729 (23.50) | 30.795 (17.98) | 0.33 |
| BV6 | 51.444 (32.67) | 46.465 (26.80) | 0.29 | 45.144 (28.53) | 38.904 (22.19) | 0.19 |
| BV7 | 62.58 (38.64) | 57.08 (32.76) | 0.42 | 55.094 (33.62) | 48.022 (27.02) | 0.34 |
| BV8 | 73.311 (43.17) | 66.957 (37.05) | 0.31 | 64.55 (37.75) | 56.50 (30.94) | 0.18 |
| BV9 | 83.269 (47.56) | 75.473 (40.16) | 0.15 | 73.369 (41.51) | 63.898 (33.75) | 0.13 |
| BV10 | 92.414 (50.71) | 84.953 (45.02) | 0.26 | 81.651 (44.56) | 72.145 (37.43) | 0.15 |
| BV11 | 102.387 (55.68) | 93.693 (48.46) | 0.14 | 90.72 (48.52) | 79.926 (40.80) | 0.15 |
| BV12 | 110.937 (58.45) | 101.737 (50.78) | 0.14 | 98.333 (50.77) | 86.803 (42.59) | 0.10 |
| BV13 | 118.482 (60.95) | 108.413 (53.09) | 0.08 | 105.214 (53.28) | 92.757 (44.63) | 0.08 |
| BV14 | 125.053 (63.05) | 115.01 (55.44) | 0.12 | 111.276 (55.13) | 98.631 (46.51) | 0.10 |
| BV15 | 130.481 (64.81) | 120.102 (56.97) | 0.11 | 116.297 (56.75) | 103.226 (47.96) | 0.09 |
| BV16 | 135.022 (66.05) | 124.333 (58.15) | 0.13 | 120.506 (57.93) | 107.001 (49.01) | 0.10 |
| BV17 | 138.477 (67.03) | 127.644 (58.96) | 0.13 | 123.718 (58.73) | 109.96 (49.72) | 0.09 |
| BV18 | 141.540 (67.72) | 130.362 (59.78) | 0.10 | 126.601 (59.51) | 112.422 (50.54) | 0.07 |
| BV19 | 143.837 (68.43) | 132.506 (60.34) | 0.09 | 128.753 (60.18) | 114.33 (50.89) | 0.06 |
| BV20 | 145.701 (68.80) | 134.23 (60.82) | 0.08 | 130.544 (60.58) | 115.898 (51.29) | 0.06 |
| **TBV** | **153.969 (69.78)** | **141.339 (61.93)** | **0.04** | **138.332 (61.95)** | **123.08 (52.68)** | **0.03** |

# References

1. Chen YH, Shi F, Christodoulou AG, Xie YB, Zhou ZW, Li DB. Efficient and Accurate MRI Super-Resolution Using a Generative Adversarial Network and 3D Multi-level Densely Connected Network. Medical Image Computing and Computer Assisted Intervention - Miccai 2018, Pt I. 2018;11070:91-9.

2. Ledig C, Theis L, Huszár F, Caballero J, Cunningham A, Acosta A, et al., editors. Photo-realistic single image super-resolution using a generative adversarial network. Proceedings of the IEEE conference on computer vision and pattern recognition; 2017.

3. Kim T, Kim WJ, Lee CH, Chae KJ, Bak SH, Kwon SO, et al. Quantitative computed tomography imaging-based classification of cement dust-exposed subjects with an artificial neural network technique. Computers in biology and medicine. 2022;141:105162-.

4. Park J, Kim S, Lim JK, Jin KN, Yang MS, Chae KJ, et al. Quantitative CT image-based structural and functional changes during asthma acute exacerbations. Journal of Applied Physiology. 2021;131(3):1056-66.

5. Ho TT, Kim T, Kim WJ, Lee CH, Chae KJ, Bak SH, et al. A 3D-CNN model with CT-based parametric response mapping for classifying COPD subjects. Scientific Reports. 2021;11(1):34.

6. Soft C. AVIEW COPD [Available from: <https://www.corelinesoft.com/en/solutions/copd>].
